# Lycopene Supplemented Mediterranean Diet Ameliorates Experimental Autoimmune Encephalomyelitis (EAE) in Mice and Changes Intestinal Microbiome

**DOI:** 10.1007/s11481-025-10212-7

**Published:** 2025-05-05

**Authors:** Tutku Atuk Kahraman, Müge Yılmaz, Kübra Aslan, Halit Canatan, Ayca Kara, Ozkan Ufuk Nalbantoglu, Aycan Gundogdu, Ahmet Eken

**Affiliations:** 1 Department of Nutrition and Dietetics, Institute of Health Sciences, Erciyes University, Kayseri, 38039 Türkiye; 2 Department of Nutrition and Dietetics, Faculty of Health Sciences, Erciyes University, Kayseri, 38030 Türkiye; 3 Department of Medical Biology, Faculty of Medicine, Erciyes University, Kayseri, 38030 Türkiye; 4 Genome and Stem Cell Center (GenKok), Erciyes University, Melikgazi, Kayseri 38280 Türkiye; 5 Department of Computer Engineering, Faculty of Engineering, Erciyes University, Kayseri, 38030 Türkiye; 6 Department of Microbiology and Clinical Microbiology, Faculty of Medicine, Erciyes University, Kayseri, 38030 Türkiye; 7 Department of Immunology and Microbiology, University of Colorado Anschutz Medical Campus, Aurora, CO USA; 8 Current Address: 6/b, 2404th Street, Yenişehir, Mersin, 33110 Türkiye

**Keywords:** Multiple sclerosis, Experimental autoimmune encephalomyelitis, Inflammation, Lycopene, Mediterranean diet, Microbiome

## Abstract

This study aimed to determine the effects of the Mediterranean diet (MD) and lycopene on the development of EAE and on inflammatory markers. In the 43-day study, 72 female C57BL/6 mice were randomly divided into eight groups according to whether they were EAE or naive (control) mice, fed a Western diet or a MD, and whether they received lycopene. During the study, mice were fed ad libitum, and lycopene groups were given 10 mg/kg/day lycopene per mouse every other day for 28 days in oral gavage. The mice were scored for EAE, sacrificed and their spleen, lymph nodes, and spinal cords were removed. We observed slightly delayed EAE onset in the MD-Lyc group compared to the others, and the EAE clinical scores were also lower than in the other groups. T-cell counts in the spleen and lymph nodes of the MD-Lyc group were significantly lower than in other groups. The production of IFN-γ and IL-22 was higher than in the other groups. IL-17 A cytokine produced in the spleen was lower in the MD-Lyc group than in the other groups. In addition, the highest myelination score was seen in the MD-Lyc group. MD-Lyc group also had a unique microbiome profile compared with the remaining groups. In summary, MD and lycopene administration positively impacted EAE scores and myelination. However, more comprehensive studies at the in vitro and in vivo levels are needed to reveal the effect of this intervention on cell numbers in the CNS.

**Graphical Abstract:**

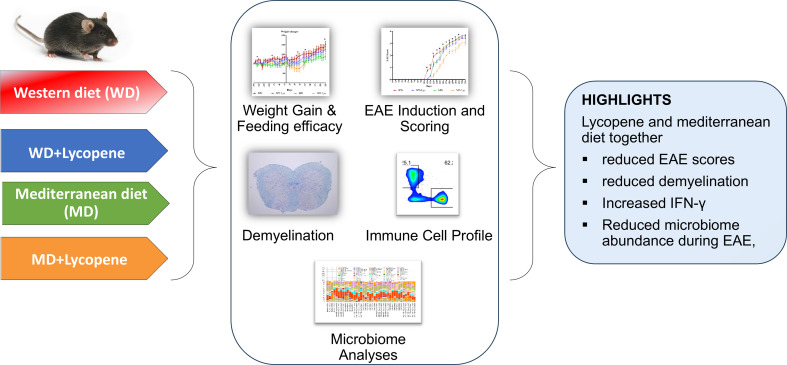

**Supplementary Information:**

The online version contains supplementary material available at 10.1007/s11481-025-10212-7.

## Introduction

Multiple Sclerosis (MS) is a chronic demyelinating autoimmune disease of the Central Nervous System (CNS), characterized by myelin loss, inflammation, and ultimately neurodegeneration (Stoiloudis et al. 2022). As a result of myelin loss, disruptions occur in the transmission of electrical impulses across neurons leading to symptoms including blurred vision, tingling sensations, dizziness, and fatigue (MSIF 2020). The estimated number of people with MS worldwide reached 2.8 million in 2020 (MSIF 2020). This number is 30% higher than estimated in 2013. The global prevalence of MS in 2020 is 35.9 per 100,000 people. The prevalence of MS has increased in all regions of the world since 2013 (Walton et al. 2020).

Multiple Sclerosis is a heterogeneous disease with clinical and pathological features that involve different pathways leading to tissue damage, whereas the cause of MS is not fully understood. The most widely accepted theory is that MS begins as an inflammatory, immune-mediated disease characterized by autoreactive lymphocytes (Thompson et al. 2018). The disease is then dominated by microglial activation and chronic neurodegeneration (Olek and Mowry 2024). The cellular immunology of MS consists of interactions between T cells, B cells, myeloid cells, and other immune system cells (Bar-Or and Li 2021). Current pharmacological treatments approved for patients reduce the frequency of attacks by suppressing or eliminating autoreactive immune cells and thus have an impact on lesion development (Attfield et al. 2022). However, its effects on pathological processes associated with disease progression are not clearly identified (Attfield et al. 2022).

Dysfunction of the immune system is present in neuroinflammatory diseases including MS, and changes in the microbiota can affect the function of the immune system in various ways (Correale et al. 2022). Mechanistically, microbiota could modulate intestinal permeability in MS patients; play a protective role in the integrity of the blood-brain barrier; microbial dysbiosis, thus, and could affect MS pathogenesis (Wang et al. 2022). In this regard, Miyake et al. 2015 found that *Bifidobacterium* and *Streptococcus* species were enriched and *Bacteroides*,* Faecalibacterium*,* Prevotella*, and *Anaerostipes* species were reduced in the microbiota of Relapsing Remitting MS (RRMS) patients compared to healthy controls. Chen et al. 2016 reported increase in the genera *Pseudomonas*,* Pedobacter*,* Mycoplasma*,* Haemophilus*,* Blautia*, and *Dorea* and decrease in the *Parabacteroides*,* Adlercreutzia*,* Collinsella* and *Lactobacillus* genera in RRMS patients. Jangi et al. 2016 reported that *Methanobrevibacter* and *Akkermansia* levels increased and *Butyricimonas* levels decreased in RRMS patients. Primary progressive MS (PPMS) has been associated with reductions in Butyricococcus, which is known to produce short chain fatty acids (SCFAs) and can therefore mediate anti-inflammatory effects by inducing Treg cells (Reynders et al. 2020).

The nutrition has been known to play an important role in modulating MS pathogenesis, thus in addition to pharmacological therapy, nutrition or lifestyle recommendations may support MS treatment (Stoiloudis et al. 2022). Certain dietary components may reduce CNS inflammation and regulate immunity through microbiota-dependent/independent mechanisms or by affecting blood-brain barrier permeability (Valburg et al. 2021). In addition to neuroinflammation, neurodegeneration is also present in the very early stages of MS. Increased oxidative stress can induce damage to the cellular structure and potentially destroy tissues. Inflammation could contribute to the reactive oxygen species (ROS) and reactive nitrogen species (RNS) production, and vice versa, establishing an auto-toxic loop that helps develop a pathophysiologic mechanism in diseases like MS. Activated microglia is the principal ROS producer (Simpson and Oliver. 2020). High amounts of ROS could act over mitochondria impeding the ATP production necessary for neurons and glia’s regular activity. Furthermore, RSN can induce excitotoxicity through the glutamate release and impair its uptake system. The inflammasome contributes to the establishment of an inflammatory state, releasing pro-inflammatory cytokines and increasing oxidative stress. All of these mechanisms participate in the development and progress of MS (Ramos-González et al. 2024). Therefore, studies revealed that dietary components that reduce ROS, improve mitochondrial function, and promote myelin repair and neuroprotection is indeed beneficial for MS (Mastronardi et al. 2004; Van Horssen et al. 2011; Yoon et al. 2016; Valburg et al. 2021). Vitamins A and D, polyunsaturated fatty acids (PUFA), and polyphenols found in the diet can reduce inflammation by regulating the activation of inflammatory cells and reduce oxidative stress, thus preventing chronic demyelination and axonal damage (Stoiloudis et al. 2022). High-salt diet has also been shown to negatively impact MS pathology (Zostawa et al. 2017).

One of the dietary models whose effects on MS have been investigated is the Mediterranean Diet (MD) (Atabilen and Akdevelioğlu 2023). MD is a sustainable dietary model in which the main source of fat is olive oil which is rich in monounsaturated fatty acids (MUFA) and polyphenols, and in which plant foods such as grains, vegetables, fruits, and legumes are frequently consumed. In this diet, consumption of red meat, processed meat and sweets is limited (Serra-Majem et al. 2020). Foods and foodstuffs such as olive oil, polyphenols, phytochemicals, omega-3 (n-3) fatty acids, and fiber, which are frequently consumed in the MD, show antioxidant and anti-inflammatory effects through various mechanisms (Tosti et al. 2018; Barrea et al. 2020; Barbouti and Goulas 2021; Itsiopoulos et al. 2022). When the effect of MD on individuals with MS was examined, a significant negative relationship was found between compliance with MD and the Expanded Disability Status Scale (EDSS) score (Esposito et al. 2021). It has also been reported that there is a reduced risk of CNS demyelination in MS patients with high compliance with MD (Black et al. 2019a, b). Additionally, MD-style eating is associated with fewer attacks (Moravejolahkami et al. 2020a). In studies using dietary intervention, it was found that MD provided a significant improvement in the fatigue scale score (Katz Sand et al. 2019; Moravejolahkami et al. 2020b; Razeghi-Jahromi et al. 2020) and an increase in quality of life (Moravejolahkami et al. 2020b). Although there is no study showing the effect of MD on Experimental Autoimmune Encephalomyelitis (EAE), there are studies showing that a diet low in total and saturated fat improves EAE (Ahn et al. 2014) and that MD components have positive effects on EAE (Unoda et al. 2013; Haghikia et al. 2015; Mancera et al. 2017; Conde et al. 2020).

Lycopene is one of the components of the MD and is found mostly in tomatoes and other vegetables and fruits (Caseiro et al. 2020; Di Mascio et al. 1989). Lycopene is an acyclic isomer of β-carotene which lacks provitamin A activity due to its ionic ring structure (van Steenwijk et al. 2020). However, lycopene is among the most potent antioxidants, with a singlet-oxygen quenching ability that is two times higher than β-carotene and ten times higher than alpha-tocopherol (van Steenwijk et al. 2020). It shows anti-inflammatory and antioxidant effects by reducing pro-inflammatory cytokines and ROS accumulation and has neuroprotective properties with yet untested potential for MS treatment as a supplement (Qu et al. 2011; Caseiro et al. 2020; Paul et al. 2020). Lycopene has the ability to reduce reactive oxygen species (ROS) and eliminate singlet oxygen, nitrogen dioxide, hydroxyl radicals, and hydrogen peroxide. Its effect on reactive oxygen species includes radical attachment, electron transfer, and allylic hydrogen abstraction (Kulawik et al. 2023).

Most of the studies examining the relationship between the MD and MS are studies that evaluate compliance with MD and do not involve dietary intervention. In addition, although lycopene has been shown to have protective effects in various inflammatory and neurodegenerative diseases (Caseiro et al. 2020; Paul et al. 2020), no study has been found investigating the effects of lycopene on MS and EAE. Unlike previous studies, this study aimed to determine the effects of MD and lycopene on the development of EAE and on inflammatory markers.

## Materials and Methods

### Mice

Seventy-two 6–8 weeks old C57BL/6 female mice were purchased from Radon Medikal Ltd. Şti (http://www.radonltd.com/) and used in this study. The mice were acclimatized for two weeks in the vivarium prior to use at an average temperature of 22–24 °C, 55–60% relative humidity, 12 h of darkness, and 12 h of light. During this period, mice were fed *ad libitum*. This study was approved by the Erciyes University Animal Experiments Local Ethics Committee at the meeting dated 08.09.2021 with number 08 and decision number 21/174.

### Diet and Lycopene Intervention

At the end of the acclimation period, the mice were divided into 8 different intervention groups: EAE mice that were fed Western diet (WD/EAE) (*n* = 12), EAE mice that were fed Western diet and administered lycopene (WD-Lyc/EAE)(*n* = 12), EAE mice that were fed Mediterranean diet (MD/EAE) (*n* = 12), EAE mice that fed Mediterranean diet and administered lycopene (MD-Lyc/EAE) (*n* = 12), naive mice that were fed Western diet (WD/Naive) (*n* = 6), naive mice that fed Western diet and administered lycopene (WD-Lyc/Naive) (*n* = 6), naive mice that were fed Mediterranean diet (MD/Naive) (*n* = 6), naive mice that were fed Mediterranean diet and administered lycopene (MD-Lyc/Naive) (*n* = 6). The experimental procedure is shown in Supplementary File 1 and the intervention diets in Supplementary Tables 1–2 (Vang et al. 2011; Davis et al. 2015; Bernstein et al. 2016; Husted and Bouzinova 2016; Weiskirchen and Weiskirchen 2016; Barrington et al. 2018; EFSA et al. 2018).

When evaluating nutritional intake, total food consumption was calculated and divided by the number of mice in the cage. Food intake and feeding efficiency were calculated with the following formula (Wu et al. 2019): Food intake = [initial food weight - remaining food weight (g)]. Feeding efficiency = [body weight gain (g)] / [average nutritional intake (g)]. The calculated feeding efficiency was expressed as a percentage by multiplying by 100.

Lycopene with 96% purity in powder form (CAS: 502-65-8), was purchased from Henan Tianfu Chemical Co.Ltd (www.tianfuchem.net). Lycopene was dissolved in sunflower oil, and given by oral gavage at a dose of 10 mg/kg/day per mouse every other day for 28 days based on previous studies (Prema et al. 2015; Bhardwaj and Kumar 2016).

### EAE Induction

For EAE induction, MOG_35 − 55_ peptide (ChinaPeptides) was prepared in 1 mg/ml Phosphate Buffer Solution (PBS). Complete Freund’s Adjuvant (CFA) (Sigma, cat: 344289-1SET) 4 mg/ml was fortified with the addition of inactivated desiccated *Mycobacterium Tuberculosis* H37Ra (Difco, cat: DF3114-33-8). The emulsion was prepared by mixing Peptide solution with equal volume of fortified CFA with via a three-way stopcock T-connector. The prepared emulsion was injected subcutaneously into the right and left hip/abdomen junction areas of the mice, 100 µl to each site. Additionally, on the day of immunization (day 0) and the day 2, Pertussis Toxin was injected intraperitoneally at 200 ng/mice dose in 200 µl of PBS. (Eken et al. 2021). A total of 48 mice in the EAE groups were immunized, monitored daily for the development of EAE, and the disease was scored by the researchers according to criteria (Hooke Laboratories 2008–2023).

### Isolation of Leukocytes from Spleen and Lymph Nodes

Mice were anesthetized with isoflurane and perfused with 30 mL of 1X PBS at the left ventricle. Inguinal lymph nodes and spleen were harvested in 1X sterile PBS solution. Spleens were mascerated with the back of the syringe mechanically and filtered through a 70-µm strainer and spun at 300 g for 5 min. After the centrifuge, supernatant was removed and the pellet dissolved in ACK Lysis buffer (1 ml for each spleen) to remove erythrocytes and centrifuge again at 300 g for 5 min. The cells were washed with 1X PBS twice. The cells were counted on the hematocytometer with Trypan Blue and used. Inguinal lymph nodes were processed similarly without ACK lysis (Eken et al. 2017, 2021).

#### Cell Surface Staining

One million cell/mice were seeded in 96 well plate and blocked with Fc-block in 200 µl staining buffer (2%FBS with PBS) for 5 min on ice. The antibodies were added according to manufacturer’s guidelines and stained for 30 min in dark at 4ºC, and washed twice with staining buffer and run on FACSAria III. To measure the absolute number of CD4^+^ and CD8^+^ cells, Spherotech beads (Cat No ACBP-100-10) were added onto cells at recommended dilution (Yilmaz et al. 2022; Odabas et al. 2024). Cells were stained with the following anti-mouse antibodies: B cell mix: Anti mouse CD19-FITC (Clone:1D3/CD19), Anti-mouse CD138-PerCp/Cy5.5 (Clone:281-2), Myeloid lineage mix: Anti-mouse CD11b-APC (Clone: M1/70), Anti-mouse Ly6G-PE/Cy7 (Clone:1A8), T cell mix: Anti-mouse CD3-FITC (Clone:45-2C11), Anti-mouse CD4-PE (Clone: GK1.5), Anti-mouse CD8-PE/Cy7 (Clone: SK1).

#### Transcription Factor FOXP3 Staining

Cells were surface stained as described above. Then, the cells were fixed and permeabilized according to the manufacturer’s instructions (FOXP3 True Nuclear Transcription Factor Kit (Biolegend-424401). After fixation and permeabilization, cells were stained anti-mouse FOXP3-Alexa Fluor 647 (Clone: MF-14) antibody for 30 min on ice in dark. Then the cells were washed staining buffer twice and analyzed on FACSAria III (Kasap et al. 2022).

#### Intracellular Staining

Cells (1 × 10^5^) were seeded into 96-well U bottom plate and were stimulated with PMA (50 ng/ml), Ionomycin (1 µg/ml), Golgi Plug (1 µg/ml) for 4 h at 37ºC in 0.05% CO_2_ incubator. After the stimulation, cells were surface stained with T cell antibodies as described above. After surface staining, cells were fixed with 200 µl BD-ICC Fixation buffer and incubated on ice for 20 min. Cells were centrifuged at 400 g for 3 min and washed twice with 150 µl BD-ICC permeabilization buffer. The cells were stained with following the antibodies and acquired run on FACSAria III as described above (Eken et al. 2017, 2021). ICC mix: Anti-mouse GM-CSF-Pacific Blue (Clone: MP1-22E9), Anti-mouse IL-22-PerCp/Cy5.5 (Clone: Poly5164), Anti-mouse IFN-γ-Brilliant Violet 510 (Clone: XMG1.2), Anti-mouse IL-17 A-APC (Biolegend) (Clone: TC11-18H10.1).

### Histological Procedure

Spinal cord tissues obtained from mice were fixed in 4% formaldehyde for 72 h. Then, they were washed in running tap water for 1 h and then passed through an increasing series of alcohol to dehydrate them. Afterward, they were treated with xylene and embedded in paraffin. Sections were taken from the tissues obtained from the blocks and stained with Luxol fast blue. 4x and 20x size photographs were taken with a Leica microscope and density measurements were made with the Image J program (Aydin et al. 2022).

### Microbiome Analyses

The sequencing data were analyzed using the QIIME2 pipeline (Bolyen et al. 2019), after the reads were filtered and trimmed to achieve a PHRED quality score of 30 using the Trimmomatic tool (Bolger et al. 2014). Amplicon Sequence Variants (ASVs) were identified through the DADA2 method. Following this, the representative sequences of the ASVs were categorized into taxonomic clades using the Naïve Bayesian Classifier, which had been trained on the Silva database (version 132) (Quast et al. 2013). The evaluation of alpha diversity statistics was also carried out using scripts from the QIIME2 pipeline. To quantify the alpha diversity of each sample, Shannon and Chao1 indices were employed. For statistical analysis, the Mann–Whitney U test was applied. Additionally, the false discovery rate correction of p-values was conducted using the Benjamini–Hochberg procedure.

### Statistical Analyses

Flow cytometry graphs were evaluated with the help of FlowJo. All data were analyzed on GraphPad Prism 9 program. Normality of data was checked by the Shapiro-Wilk test. ANOVA and Tukey’s tests were used for normally distributed multiple comparisons, and data are presented as mean ± standard error (X ± SEM). In multiple comparisons that were non-normally distributed, Kruskal Wallis Test and Dunn’s correction were used and the data are given as median (interquartile range) (M//IQR). The p value less than 0.05 was considered statistically significant (Eken et al. 2021; Odabas et al. 2024).

## Results

### Weight Change, Feeding Efficiency, and Differences in Inflammation Markers of Naive Mice

**Fig. 1:**
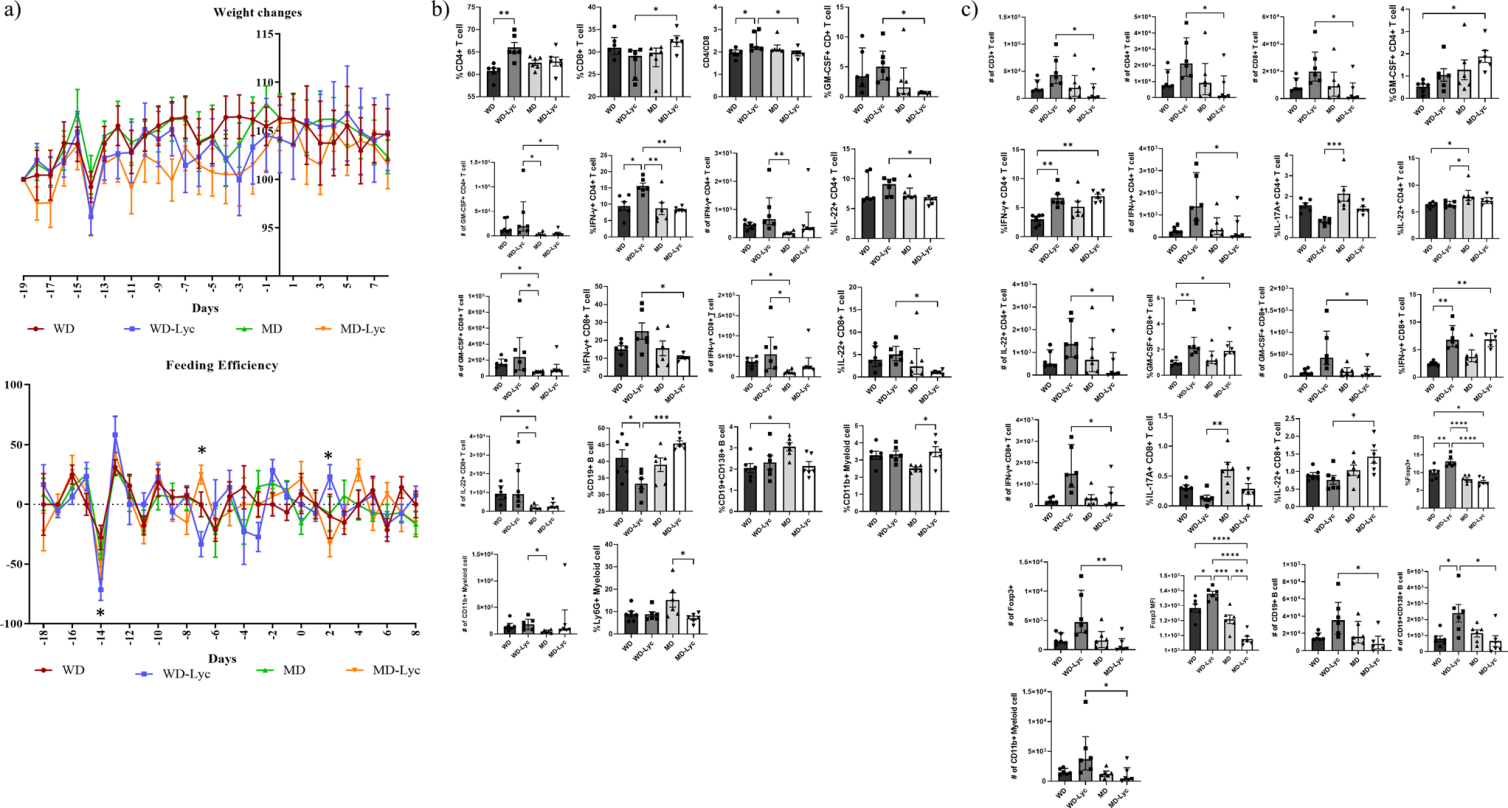
**(a)** Weight changes and feeding efficiency of naive mouse groups over time. **(b)** Examining the effect of the intervention on immune cells in the spleens of naive mice. **(c)** Examining the effect of the intervention on immune cells in the lymph nodes of naive mice. *p < 0.05; ** *p* < 0.005; ***p < 0.001; ****p < 0.0001

The change in weight of naive mice throughout the experimental procedure did not show a statistically significant difference between the groups (*p* > 0.05). Similarly, the feeding efficiency was comparable between the groups across 27 day period except three timepoints (on the − 14th day (WD and WD-Lyc, *p* = 0.041), on the − 7th day (WD-Lyc and MD-Lyc, *p* = 0.007), and on the 2nd day (WD-Lyc and MD-Lyc, *p* = 0.015) (Fig. 1.a).

The impact of diet and lycopene on immune cell profiles differed between spleen and lymph nodes. In spleens of naïve mice (Fig. 1.b, Supplementary Table 3), CD4 + T cell frequency was significantly higher in the WD-Lyc group (*p* = 0.002), CD8 + T cell frequency was higher in MD-Lyc (*p* = 0.005), and the CD4/CD8 ratio increased in WD-Lyc (*p* < 0.05). GM-CSF + CD4 + T cell percentage and count were significantly higher in WD-Lyc compared to MD-Lyc, MD (*p* = 0.022, *p* < 0.05). IFN-γ + CD4 + T cell frequency was significantly elevated in WD-Lyc vs. WD (*p* = 0.012), MD (*p* = 0.005), and MD-Lyc (*p* = 0.003), with counts also higher than in MD (*p* = 0.001). IL-22 + CD4 + T cell frequency was higher in WD-Lyc vs. MD-Lyc (*p* = 0.025). GM-CSF + CD8 + T cell numbers were significantly increased in WD-Lyc compared to WD and MD (*p* < 0.05). IFN-γ + and IL-22 + CD8 + T cell frequencies were higher in WD-Lyc vs. MD-Lyc, with cell numbers elevated in WD and WD-Lyc vs. MD (*p* < 0.05). CD19 + cell frequency was higher in WD and MD-Lyc groups compared to WD-Lyc. CD19 + CD138 + plasma cell percentage was elevated in MD vs. WD (*p* < 0.05). Among myeloid cells, CD11b + percentage was higher in MD-Lyc vs. MD (*p* = 0.024), and counts were greater in WD-Lyc vs. MD (*p* < 0.05). Ly6G + cell frequency was higher in MD than in MD-Lyc (*p* = 0.03) (Fig. 1.b).

In contrast, CD3+, CD4+, and CD8 + T cell numbers in lymph nodes were significantly higher in WD-Lyc vs. MD-Lyc (*p* = 0.033, *p* = 0.033, *p* = 0.029) (Fig. 1.c, Supplementary Table 4). GM-CSF + CD4 + T cell frequency was greater in MD-Lyc vs. WD (*p* = 0.022). IFN-γ + CD4 + T cell percentage was higher in WD-Lyc and MD-Lyc vs. WD (*p* < 0.005), with higher cell counts in WD-Lyc vs. MD-Lyc (*p* = 0.033). IL-17 A + CD4 + T cell frequency was elevated in MD vs. WD-Lyc (*p* < 0.001), while IL-22 + CD4 + T cell frequency was higher in MD vs. WD-Lyc and WD, and IL-22 + CD4 + T cell counts were greater in WD-Lyc vs. MD-Lyc (*p* < 0.05, *p* = 0.037). GM-CSF + and IFN-γ + CD8 + T cell frequencies were higher in WD-Lyc and MD-Lyc vs. WD, with higher counts in WD-Lyc vs. MD-Lyc (*p* < 0.05). IL-17 A + CD8 + T cell frequency was elevated in MD vs. WD-Lyc (*p* = 0.003), and IL-22 + CD8 + T cells were more frequent in MD-Lyc vs. WD-Lyc (*p* = 0.015). Foxp3 + Treg frequency, cell number, and MFI were highest in WD-Lyc and lowest in MD-Lyc (*p* < 0.0001, *p* = 0.006, *p* < 0.0001). CD19 + B cell numbers were higher in WD-Lyc vs. MD-Lyc, CD19 + CD138 + plasma cells were elevated in WD-Lyc vs. WD and MD-Lyc, and CD11b + cell numbers were higher in WD-Lyc vs. MD-Lyc (*p* < 0.05) (Fig. 1.**c**). Collectively, these findings suggest lycopene boosts CD3+, CD4+, and CD8 + T cells in lymph nodes and enhances IFN-γ production, especially when combined with WD and MD in lymph nodes, and with WD in spleen.

### Weight Change, Feeding Efficiency, EAE Incidence, EAE Clinical Score, and Histological Findings of EAE Mice

**Fig. 2:**
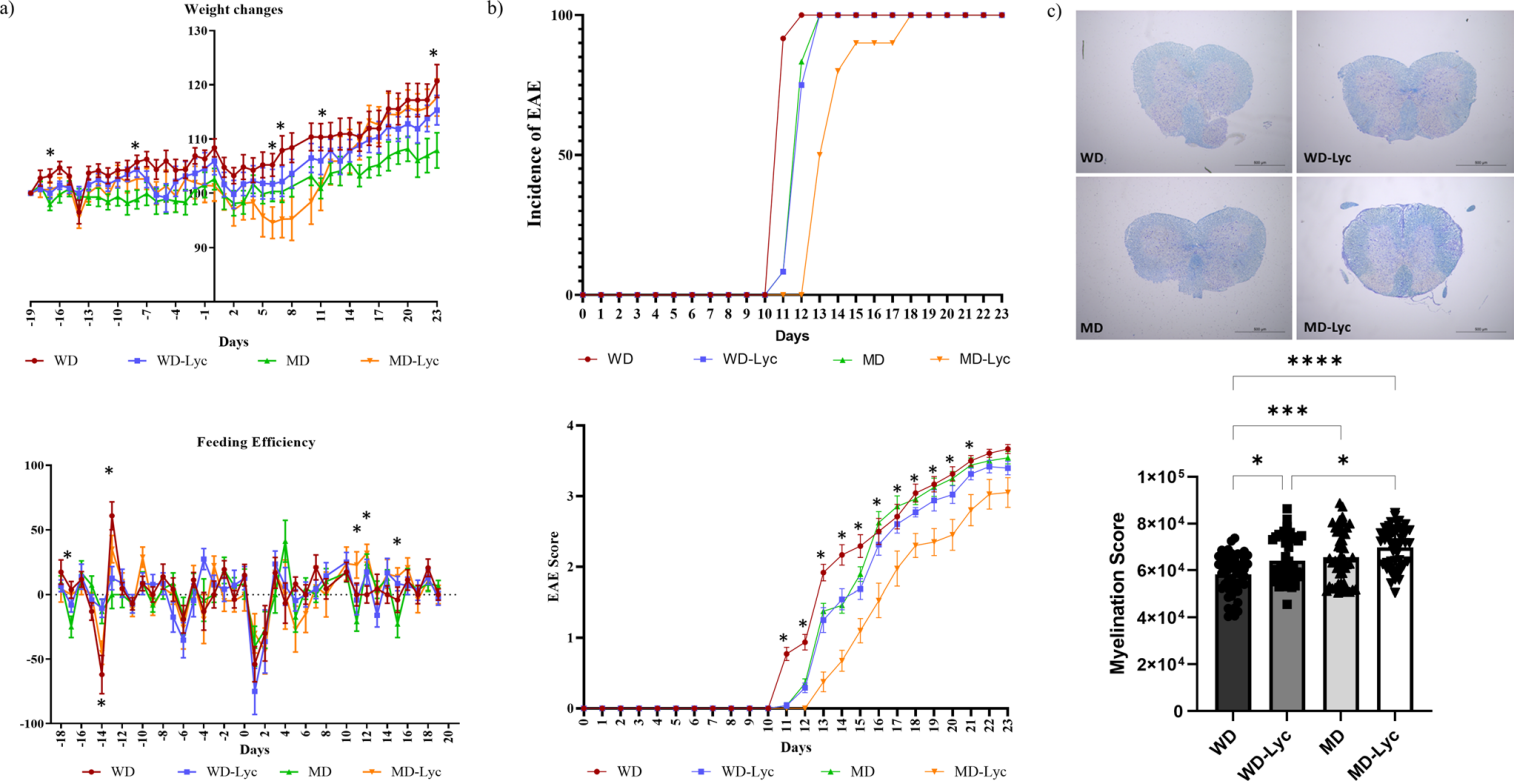
**(a)** Weight changes and feeding efficiency of EAE mouse groups over time. **(b)** Incidence of EAE in mice and clinical scoring. **(c)** Examining the effect of the intervention on myelination in the spinal cords of EAE mice. *p < 0.05; ***p < 0.001; ****p < 0.0001

In EAE mice throughout the experimental procedure, the rate of change in weight of the WD group was found to be statistically significantly higher than the MD group on day − 17, day − 8, day 11, and day 23 (*p* = 0.026, *p* = 0.018, *p* = 0.031, *p* = 0.042, respectively) and reflected as weight gain. On the 6th and 7th days, it was significantly higher in the WD group than in the MD-Lyc group (*p* = 0.040, *p* = 0.044, respectively). The feeding efficiency of EAE mice was higher in the MD-Lyc group than in the MD group on day − 17, day 11 and day 15; on the − 14th day, in the WD-Lyc group, compared to the WD and MD-Lyc groups; on the − 13th day, in the WD group, compared to the WD-Lyc and MD groups; on the 12th day, it was found to be significantly higher in the MD-Lyc group than in the WD group (*p* < 0.05) (Fig. 2.a).

The incidence of EAE did not differ significantly between the groups. However, the clinical score of the WD group on the 11th day was significantly higher than the other groups (*p* < 0.0001). While the difference between the WD group and the other groups continued on the 12th, 13th, and 14th days, statistically significant differences also occurred between the WD-Lyc and MD-Lyc groups and between the MD and MD-Lyc groups (*p* < 0.05). It was found to be significantly higher in the WD-Lyc and MD groups compared to the MD-Lyc group on the 15th, 16th, 18th, 19th, and 20th days, in the MD group compared to the MD-Lyc group on the 17th day, in the WD group compared to the MD-Lyc group on the 21st day (*p* < 0.05) (Fig. 2.b).

The luxol blue staining of the spinal cords revealed that in EAE mice, the myelin staining intensity was higher in the MD-Lyc group than in the WD and WD-Lyc groups. Also, in the MD and WD-Lyc groups, it was significantly higher than the WD group (*p* < 0.05) (Fig. 2.c). Collectively, these data suggest that MD combined with lycopene has the potential to reduce the clinical disease scores as well as demyelination during MOG_35 − 55_ induced active EAE in mice.

### The Impact of Lycopene Supplemented Diet on T Cells and Cytokines in EAE Mice

**Fig. 3:**
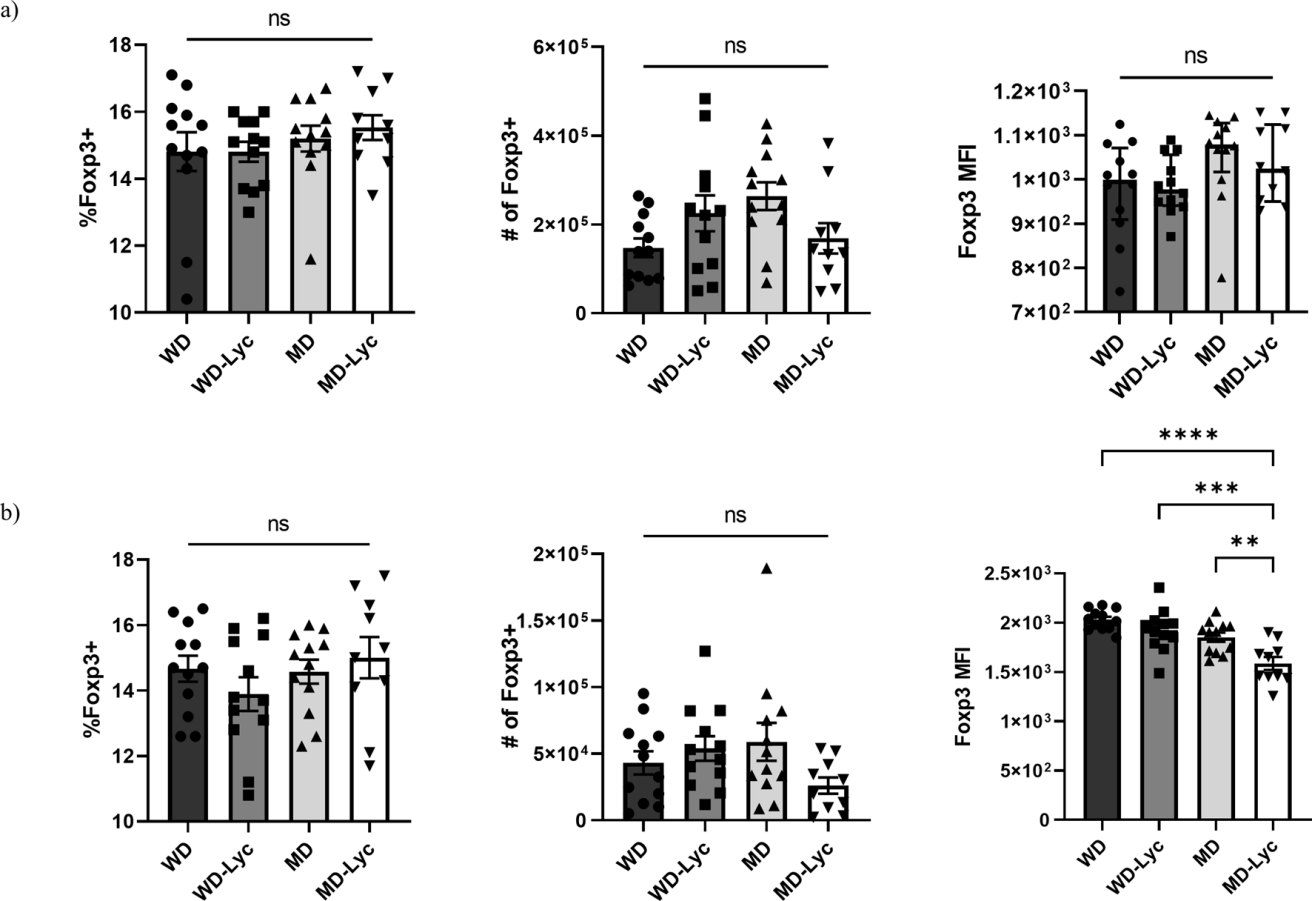
(**a**) Examining the effect of the intervention on Treg cells in the spleens of EAE mice. **(b)** Examining the effect of the intervention on Treg cells in the lymph nodes of EAE mice. ** *p* < 0.005; ***p < 0.001; ****p < 0.0001; ns: not statistically significant

There was no statistically significant difference between the groups in terms of the proportion or the number of Foxp3^+^ CD4^+^ Treg cells, nor the expression (MFI) of Foxp3 cells in the spleens of EAE mice at the peak of disease (Fig. 3.a). Similarly, when lymph nodes were examined, the Foxp3^+^ cell frequency and number were not different between the groups. The expression of Foxp3 (MFI), however, was found to be statistically significantly higher in the WD, WD-Lyc, and MD groups than in the MD-Lyc group (*p* < 0.0001, *p* < 0.001, *p* = 0.005, respectively) (Fig. 3.b).

**Fig. 4:**
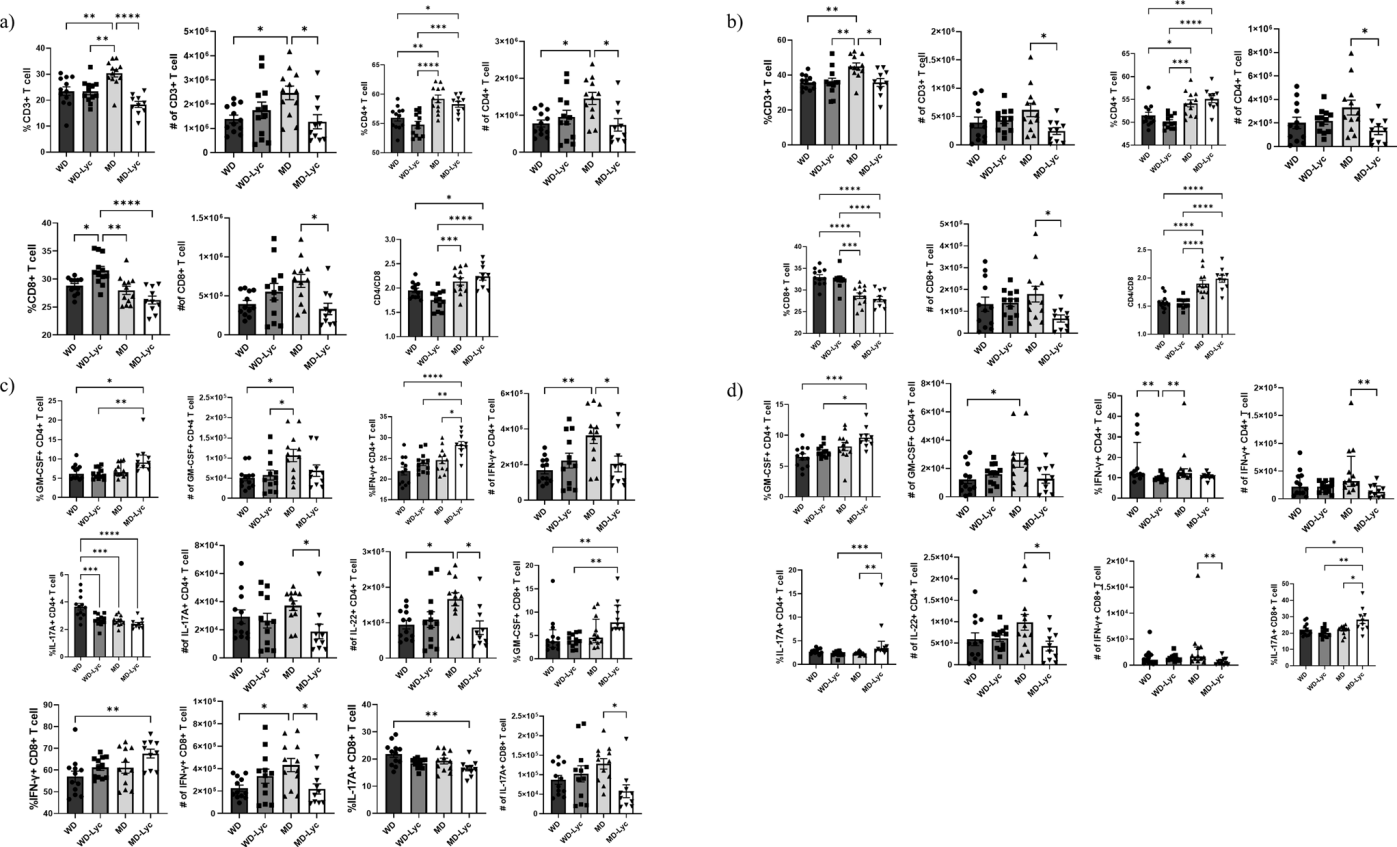
**(a)** Examining the effect of the intervention on T cells in the spleens of EAE mice. **(b)** Examining the effect of the intervention on T cells in the lymph nodes of EAE mice. **(c)** Examining the effect of the intervention on cytokine production in the spleens of EAE mice. **(d)** Examining the effect of the intervention on cytokine production in the lymph nodes of EAE mice. *p < 0.05; ** *p* < 0.005; ***p < 0.001; ****p < 0.0001

The ratio of CD3 + T cells in spleens of EAE mice was significantly higher in the MD group compared to WD, WD-Lyc, and MD-Lyc groups (*p* < 0.05). CD4 + T cell frequency was significantly higher in MD and MD-Lyc groups than in WD and WD-Lyc (*p* < 0.05), with higher CD3 + and CD4 + T cell counts in the MD group vs. WD and MD-Lyc (*p* < 0.05). CD8 + T cell percentage was elevated in the WD-Lyc group compared to others but counts differed only between MD and MD-Lyc groups (*p* < 0.05). CD4/CD8 ratio was significantly higher in the MD group vs. WD-Lyc, and in MD-Lyc vs. WD-Lyc and WD (*p* < 0.05) (Fig. 4.a, Supplementary Table 5).

In lymph nodes, CD3 + T cell frequency was significantly higher in the MD group vs. WD, WD-Lyc, and MD-Lyc (*p* = 0.007, *p* = 0.009, *p* = 0.011). CD4 + T cell frequency was higher in MD and MD-Lyc than in WD and WD-Lyc (*p* < 0.05), while CD8 + T cell frequency was higher in WD and WD-Lyc vs. MD and MD-Lyc (*p* < 0.001). CD3+, CD4+, and CD8 + T cell numbers were higher in MD vs. MD-Lyc (*p* < 0.05). CD4/CD8 ratio was significantly higher in MD and MD-Lyc than in WD and WD-Lyc (*p* < 0.0001) (Fig. 4.b, Supplementary Table 6).

GM-CSF + CD4 + T cell frequency in spleens was significantly higher in MD-Lyc vs. WD and WD-Lyc (*p* = 0.016, *p* = 0.006). GM-CSF + CD4 + T cell count was also higher in MD vs. WD and WD-Lyc (*p* < 0.05). IFN-γ + CD4 + T cell percentage was significantly higher in MD-Lyc vs. WD, WD-Lyc, and MD (*p* < 0.0001, *p* = 0.006, *p* = 0.025); cell count was higher in MD vs. WD and MD-Lyc (*p* < 0.05). IL-17 A + CD4 + T cell frequency was higher in WD vs. all other groups (*p* < 0.001), and count was higher in MD vs. MD-Lyc (*p* < 0.05). IL-22 + CD4 + T cell count was significantly elevated in MD vs. WD and MD-Lyc (*p* < 0.05). GM-CSF + CD8 + T cell percentage was higher in MD-Lyc vs. WD and WD-Lyc (*p* < 0.005). IFN-γ + CD8 + T cell frequency was elevated in MD-Lyc vs. WD (*p* = 0.009), while counts were higher in MD vs. WD and MD-Lyc (*p* < 0.05). IL-17 A + CD8 + T cell frequency was higher in WD vs. MD-Lyc (*p* = 0.003), and count was higher in MD vs. MD-Lyc (*p* < 0.05) (Fig. 4.**c**).

In lymph nodes, GM-CSF + CD4 + T cell percentage was higher in MD-Lyc vs. WD and WD-Lyc (*p* < 0.001, *p* = 0.024). GM-CSF + CD4 + T cell count was higher in MD vs. WD (*p* > 0.05). IFN-γ + CD4 + T cell frequency was higher in WD and MD vs. WD-Lyc, with higher counts in MD vs. MD-Lyc (*p* < 0.005). IL-17 A + CD4 + T cell frequency was higher in MD-Lyc vs. WD-Lyc and MD (*p* < 0.001, *p* = 0.003), and IL-22 + CD4 + T cell count was elevated in MD vs. MD-Lyc (*p* < 0.05). IFN-γ + CD8 + T cell count differed significantly in MD vs. MD-Lyc (*p* = 0.007). IL-17 A + CD8 + T cell ratio was higher in MD-Lyc vs. all other groups (*p* < 0.05) (Fig. 4.d). Overall, these findings suggest lycopene combined with MD enhances CD4 + T cells more than CD8 + T cells, increasing the CD4/CD8 ratio.

### B- and Myeloid Cells in EAE Mice Fed Lycopene Supplemented Diet

**Fig. 5:**
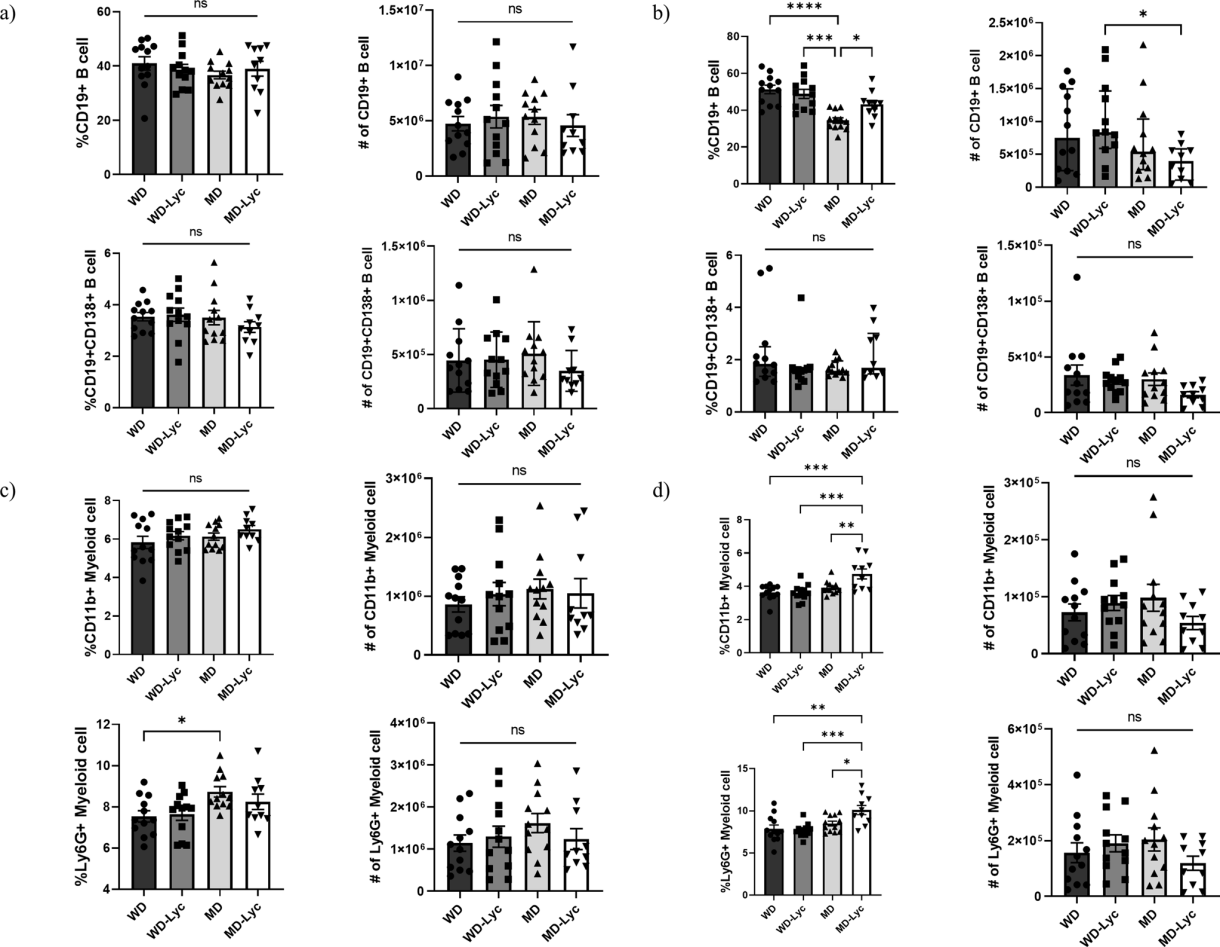
**(a)** Examining the effect of the intervention on B cells in the spleens of EAE mice. **(b)** Examining the effect of the intervention on B cells in the lymph nodes of EAE mice. **(c)** Examination of the effect of the intervention on myeloid cells in the spleens of EAE mice. **(d)** Examination of the effect of the intervention on myeloid cells in the lymph nodes of EAE mice. *p < 0.05; ** *p* < 0.005; ***p < 0.001; ****p < 0.0001; ns: not statistically significant

There was no significant difference between the groups in neither the frequency nor the number of CD19^+^ and CD19^+^CD138^+^ cells in the spleens of EAE mice (*p* > 0.05) (Fig. 5.a). When the lymph nodes were investigated, the CD19^+^ cell frequency was found to be higher in the WD, WD-Lyc, and MD-Lyc groups than in the MD group (*p* < 0.05). The number of CD19^+^ cells was significantly higher in the WD-Lyc group than in the MD-Lyc group (*p* = 0.046). The frequency and number of CD19^+^CD138^+^ cells did not show a significant difference between the groups (*p* > 0.05) (Fig. 5.b).

The Ly6G^+^ cell frequency in the spleens of EAE mice was found to be significantly higher in the MD group than in the WD group (*p* = 0.028). The frequency and number of CD11b^+^ cells and the number of Ly6G^+^ cells did not show a significant difference between the groups (*p* > 0.05) (Fig. 5.**c**). CD11b^+^ and Ly6G^+^ cell percentages were found to be significantly higher in the MD-Lyc group than in the WD, WD-Lyc, and MD groups (*p* < 0.05). The number of CD11b^+^ and Ly6G^+^ cells was not different between the groups (*p* > 0.05) (Fig. 5.d).

### The Impact of Lycopene Supplemented Diet on Intestinal Microbiota

**Fig. 6:**
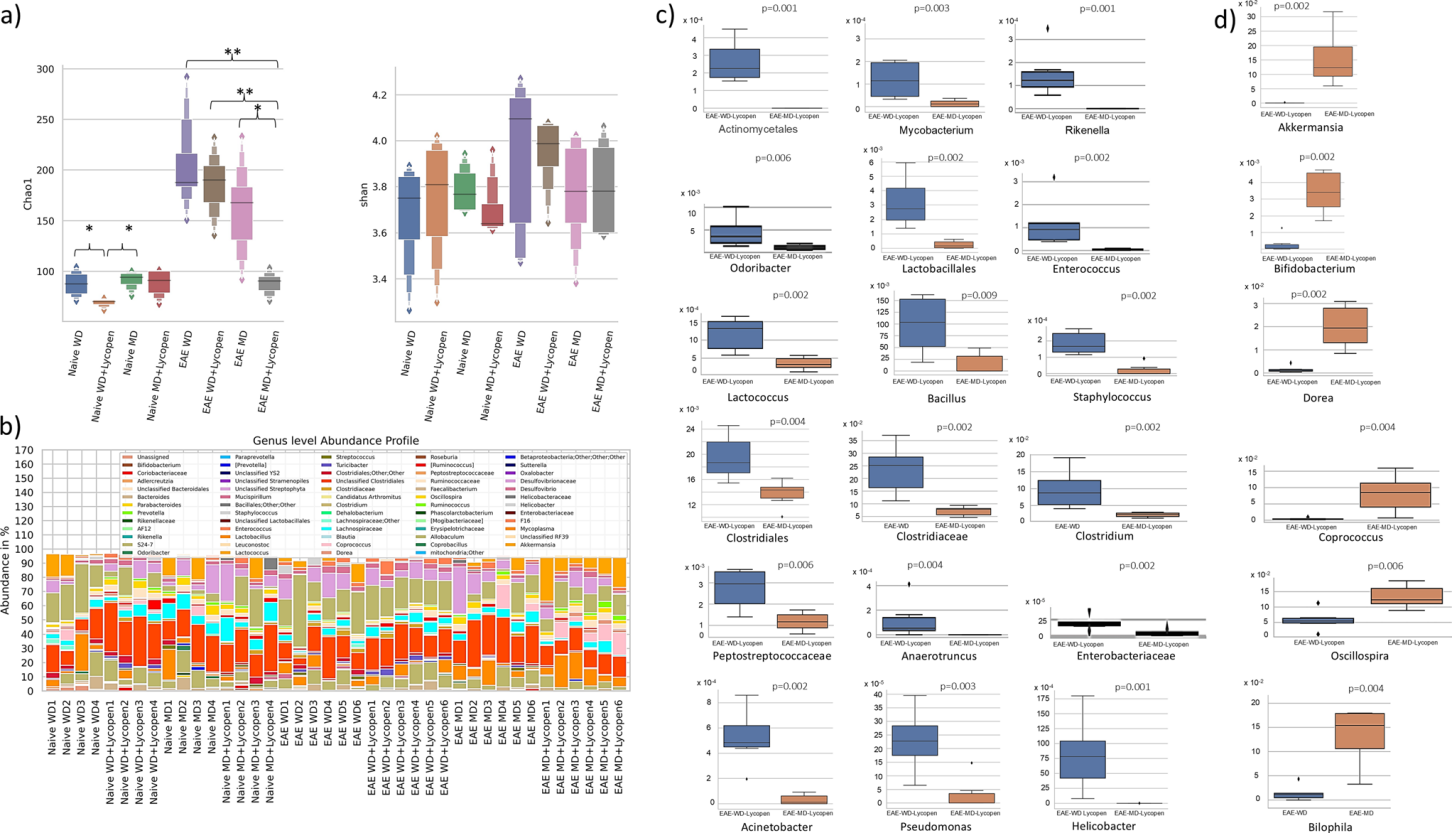
**(a)** Microbial diversity analysis profile of microbiota in mice **(b)** Genus level abundance profile of microbiota in mice. **(c)** Bacterial species that were less abundant in the microbiota of EAE mice with MD intervention. **(d)** Bacterial species that were more abundant in the microbiota of EAE mice with MD intervention. *p < 0.05; ** *p* < 0.005

In naive mice, the microbial richness of the mice fed WD and MD diet was found to be higher than the WD-Lyc group. In EAE mice, the microbial richness of WD, WD-Lyc, and MD groups was higher than in the MD-Lyc group (*p* < 0.05). Additionally, microbial richness was found to be significantly higher in EAE mice compared to naive mice (*p* < 0.05, except for comparisons of the EAE/MD-Lyc group with the Naive/WD, Naive/MD, Naive/MD-Lyc groups). Especially in EAE mice, microbiota diversity appears to be slightly decreased in MD groups compared to WD groups, but no significant difference was detected between groups in both naïve and EAE mice (*p* > 0.05) (Fig. 6.a).

When bacterial phylum and species were compared, no significant difference was found between naive mouse groups (*p* > 0.05). Additionally, no effect of lycopene was observed in the findings, so significant findings showing the effects of dietary interventions in EAE mice are included. Accordingly, in EAE groups, *Actinomycetales*,* Mycobacterium*,* Rikenella*,* Odoribacter*,* Enterococcus*,* Lactococcus*,* Bacillus*,* Staphylococcus*,* Clostridiales*,* Peptostreptococcaceae*,* Anaerotruncus*,* Enterobacteriaceae*,* Acinetobacter*,* Pseudomonas and Helicobacter* species were found to be significantly higher in the WD-Lyc group than in the MD-Lyc group. *Lactobacillales*,* Clostridiaceae* and *Clostridium* species were found to be significantly higher in the WD group than in the MD-Lyc group (*p* < 0.05). In addition, *Akkermansia*,* Bifidobacterium*,* Dorea* and *Coprococcus* species in the microbiota of the MD-Lyc group were higher than the WD-Lyc group; *Oscillospira* and *Bilophila* species were more abundant in the WD group (*p* < 0.05) (Figs. 6.c and 6.d).

## Discussion

Based on current evidence, adopting MD nutrition as a lifestyle is associated with weight loss and less body fat and reduces the risk of non-communicable diseases (Martínez-González et al. 2012; Mancini et al. 2016). Unlike saturated fat, unsaturated fats prevent weight gain by stimulating more energy expenditure, diet-induced thermogenesis and fat oxidation (Krishnan and Cooper 2014). Although participants were not given any specific advice regarding weight loss or energy count in a randomized controlled study, the weight loss of the group given MD at the end of 6 months was higher than the group without intervention (Katz Sand et al. 2019). WD causes changes (dysbiosis) in the intestinal microbiome that lead to obesity, metabolic disorders and inflammation (Zinöcker and Lindseth 2018). In our study, the weights of mice in all groups increased compared to the beginning, depending on their growth and development, and the diets given did not cause any weight loss. However, consistent with the literature, the weight change of the MD groups remained lower than the WD groups in both naïve and EAE mice until the experiment was concluded. In our study, the feeding efficiency of the mice decreased due to the oral gavage administration of lycopene and the immunization. While this situation improved over time in other groups, it continued until the day lycopene administration was stopped, especially in the MD-Lyc group in EAE mice. Overall, the mice in the MD-Lyc group gained less weight than the other groups. With the cessation of lycopene administration, the weight change in the MD-Lyc group at the end point became similar to the WD groups.

There is no study showing the effect of MD on EAE, but in studies examining its effect on individuals with MS; vegetable and fish consumption of individuals with MS has a negative effect on disability (Felicetti et al. 2022), there is a significant negative relationship between compliance with MD and EDSS score (Esposito et al. 2021), there is a decrease in disability (Katz Sand et al. 2023) and a reduced risk of CNS demyelination reported by patients with higher compliance with MD (Black et al. 2019a, b). Katz Sand et al. (Katz Sand et al. 2019), compared the MS patient groups given and not given MD, and found that the increase in EDSS in the group given MD for six months was lower than the group not given MD. In our study, there was no significant difference in the incidence of EAE between the groups throughout the experimental procedure, but the disease onset was earlier in WD, later but similar with WD-Lyc and MD, and the latest with MD-Lyc. In a study, mice were divided into groups to be fed high-fat WD or normal diet, starting two weeks before immunization and until the end of the experiment. Similar to our study after immunization, the onset of EAE in mice did not differ significantly between groups. The EAE clinical score was found to be higher in mice fed high-fat WD than in mice fed a normal diet (Timmermans et al. 2014). In our study, the EAE clinical score was generally found to be highest in the WD group and lower in the MD-Lyc group than other groups.

Ghazavi et al. (Ghazavi et al. 2019) reported that MS patients consumed less lycopene compared to the control group and that further studies are needed on this subject. Nehzat et al. (Nehzat et al. 2024) found that lycopene levels in the serum of MS patients were associated with EDSS in a 5-year follow-up. However, no intervention study showing the effect of lycopene on MS has been found in the literature to date. In our study, the WD-Lyc and MD groups were found to be similar in terms of EAE clinical score, suggesting that lycopene administration despite WD consumption may have similar effects like MD. The fact that the clinical scores of the MD-Lyc group were lower than the other groups between the 11th and 23rd days showed that the combined application of MD and lycopene was more effective than their separate application. Because lycopene is actually a component of MD, and it has been reported that not only a few specific nutrients but many foodstuffs have synergistic and interactive roles in reducing inflammation in MD (Tosti et al. 2018). These findings are thought to be due to the fact that MS is an inflammatory and neurodegenerative disease and, as mentioned in the introduction of the article, lycopene has a high ability to scavenge free radical species and has antioxidant and anti-inflammatory effects by affecting various inflammatory pathways.

A recent study showed that when mice were fed a high-fat diet rich in saturated fat, EAE scores increased, myelin staining reduced, and monocyte infiltration increased (Castro et al. 2019). Chen et al. (Chen et al. 2014) revealed in their study that demyelination decreased in mice by adding n-3 to a diet containing cuprizone, which can cause demyelination. Another study compared fat-1 genetic mice (expressing n-3 PUFA desaturase, which can endogenously synthesize n-3 from n-6 without the use of dietary supplements) and non-genetically modified wild-type mice. Mice were given a diet which was high in n-6 and low in n-3, and cuprizone for five weeks. At the end of five weeks, the degree of demyelination in the groups was found to be similar. However, the degree of remyelination two weeks after cuprizone was removed from the diet was found to be significantly higher in fat-1 mice than in wild-type mice (Siegert et al. 2017). In our study, the myelination score in the MD group of EAE mice was higher than the WD group, which supports these studies. In addition, the fact that the WD-Lyc group in the study had a myelination score similar to MD shows that lycopene is effective. Similar to other findings, the combined administration of MD and lycopene revealed the strongest effect on the myelination score. There are no studies showing the effect of lycopene on myelination in EAE mice, but other studies in animal models have shown that vitamin A plays a positive role in brain development, plasticity, remyelination, oligodendrocyte differentiation, and astrocyte responses to neuroinflammation (Fragoso et al. 2014; Mizee et al. 2014; Kim et al. 2016).

Despite publications in the literature showing that some of the MD components (such as retinoic acid, vitamin D, SCFA, Epigallocatechin gallate and n-3 fatty acids) cause an increase in Treg cells (Furusawa et al. 2013; Raverdeau et al. 2016; Wu 2016; Shahidi and Ambigaipalan 2018; Miller et al. 2019), no significant differences were found between the groups in terms of Treg numbers in our study. In addition, the mean Foxp3^+^ MFI in the lymph nodes of mice was found to be significantly lower in the MD-Lyc group than in the other groups. This result suggested that the lower disease scores in the MD-Lyc group were possibly through Treg independent mechanisms.

Timmermans et al. 2014 found significantly more CD3^+^ T cell and macrophage infiltration in the spinal cords of EAE mice fed with high-fat (21%) WD compared to mice fed with a normal diet (10% fat). In another study, mice with EAE fed with diets rich in fish oil with or without EPA were compared and it was observed that the EAE clinical score was lower and CNS infiltration of CD4^+^ T cells was reduced in mice fed a diet containing EPA (Unoda et al. 2013). In our study, T cell numbers in the spleen and lymph nodes of EAE mice were higher in the MD group than in the other groups. Additionally, the numbers of CD3^+^ and CD4^+^ T cells in the spleen were significantly lower in the WD group than in the MD group. However, when these findings are evaluated taking into account all T cells, there is CD3^+^ and CD4^+^ T cell expansion in the MD and MD-Lyc groups in both the spleen and lymph nodes, and that lycopene can stop this expansion especially in CD8^+^ T cells, and only when combined with the MD diet. It is notable that dominant population of T cell in brain lesions in MS are CD8^+^ T cells (Stojić-Vukanić et al. 2020). Although MS is a CD4^+^ T cell-driven disease, it is assumed that CD8^+^ T cells play a leading role in direct cell damage (Stojić-Vukanić et al. 2020). Based on this information, in our study, the fact that the CD4/CD8 ratio in the spleen and lymph nodes of mice with EAE was higher in the MD groups than in the WD groups and the balance was against CD8 may help explain why the EAE clinical score was generally highest in the WD group and lower in the MD-Lyc group than the other groups.

In terms of cytokine production, the only cytokine that lycopene-supplemented diets affected in the same way in both spleen and lymph node was GM-CSF, and the increasing trend stood out in the MD-Lyc group. However, due to the decrease in the number of T cells, the percent increase did not correspond to an increase in absolute numbers. IFN-γ production increased in the MD-Lyc group in both CD4^+^ and CD8^+^ T cells in the spleen, but, similarly, this was not reflected in absolute numbers due to the decrease in the number of T cells in this group. Consistent with this increase in IFN-γ in the spleen, IL-17 A production percentages also decreased in the MD-Lyc group. Similar trends were not observed for IFN-γ and IL-17 in the lymph node. It is thought that the different findings in the spleen and lymph node are due to the difference in the structure and functions of these two organs.

Studies demonstrate the critical role of GM-CSF in T cell-mediated autoimmune CNS inflammation. This cytokine can be secreted by myelin-specific activated Th1 and Th17 lymphocytes (Codarri et al. 2011; El-Behi et al. 2011). GM-CSF-deficient mice were resistant to EAE induction. While injection of this cytokine exacerbated disease symptoms, administration of blocking antibodies even after disease onset reduced the severity of the disease (McQualter et al. 2001; Codarri et al. 2011; El-Behi et al. 2011). A study showed that MS patients had significantly higher numbers of GM-CSF cells produced from CD4^+^ and CD8^+^ T cells compared to healthy controls (Rasouli et al. 2015). No study has been found showing the effects of MD or lycopene on GM-CSF. However, oleocanthal, which found in olive oil that is one of the MD components and has strong anti-inflammatory activities, has been reported to prevent the upregulation of pro-inflammatory signaling molecules such as IL-1β, IL-6, macrophage inflammatory protein-1α (MIP-1α), TNF-α and GM-CSF (Scotece et al. 2012). Although GM-CSF production in the MD groups was higher in terms of percentage production in our study, the lower disease severity may be due to the absence of an increase in absolute amount or to the fact that this cytokine increases myeloid suppressor cells.

Although IL-22 is not required for disease development in EAE mouse models, some studies have presented data demonstrating a protective role for IL-22 in EAE models (Kreymborg et al. 2007; Laaksonen et al. 2014; Lindahl et al. 2019). (Eken et al. 2021) showed that overexpression of IL-22 reduced EAE scores and demyelination, and even reduced the number of CD4^+^Th17 cells infiltrating the central nervous system. They revealed that neutralizing IL-22 did not make a significant difference in the immunopathogenesis of EAE. In our study, the number of IL-22 cells produced from CD4^+^ T cells in the spleens of EAE mice was found to be higher in the MD group than in the WD and WD-Lyc groups. In addition, considering the number and ratio of cells in the spleen and lymph nodes of mice, the highest IL-22 production from CD4^+^ T cells was detected in the MD-Lyc group. It is thought that this may be due to the fact that the main fat source of MD is olive oil. Because, although there is no study showing the effect of MD and lycopene on IL-22, it has been shown that the administration of olive oil, one of the MD components, to mice with food allergy increases the level of IL-22 and this contributed to the repair of lesions in the intestinal epithelial barrier function (Ma et al. 2023).

B cells and antibodies are known to be involved in the pathogenesis of MS, and monoclonal antibody treatments targeting B cells hold an important place in MS treatment (Gharibi et al. 2020). However, no studies showing the effects of WD and MD diets and lycopene on B cells in MS have been found. On the other hand, studies showed that the addition of n-3 PUFA to the diet in mouse models affects B cell activity (Pestka et al. 2014; Teague et al. 2014; Tomasdottir et al. 2014; Kosaraju et al. 2017; Petta et al. 2018). In this study, B cell numbers in the spleens of EAE mice were not found to be different between groups. The number of B cells in EAE mouse lymph nodes was lower in the MD-Lyc group than in the WD-Lyc group, and B cell production in the WD groups was found to be higher than in the MD groups. The proportion and number of plasma cells were not different between groups.

Myeloid lineage cells have a role in the initiation and progression of MS. In EAE and MS, the transformation of blood-borne monocytes into CNS macrophages and the activation of microglia resident in the CNS have been observed (Mishra and Wee Yong 2016). Infiltration of monocytes into the CNS is required for worsening clinical severity of EAE (Ajami et al. 2011). Additionally, neutrophil numbers increase both in the periphery and in the CNS before and at the onset of clinical EAE (Määttä et al. 1998; Nygårdas et al. 2000; Rumble et al. 2015; Pierson et al. 2018). In a previous study, monocytes from subjects fed WD exhibited increased expression of pro-inflammatory and monocyte polarization regulatory genes compared to MD (Johnson et al. 2021). The only significant finding in EAE mouse spleens in our study was that the neutrophil frequency was higher in the MD group than in the WD group. In EAE mouse lymph nodes, myeloid cell numbers are insignificantly lower in the MD-Lyc group than in the other groups, but when cell frequency is considered, MD-Lyc is the group with the highest myeloid cell production. There may be two reasons for this result: the ratio of myeloid cells in all cells increases as a result of lower T cell frequency in the MD groups, or the production of GM-CSF, which stimulates the production of myeloid cells, at a higher level in the MD groups than in the WD groups could be responsible.

The gut microbiota, though unique to each individual, is predominantly composed of *Firmicutes* (~ 60–65%), *Bacteroidetes* (~ 20–25%), *Proteobacteria* (~ 5–10%), and *Actinobacteria* (~ 3%) (Altieri et al. 2023). Diet notably influences this composition; the Western diet (WD) is linked to dysbiosis, reduced diversity, and gut permeability, while the Mediterranean diet (MD) enhances microbial biodiversity (Barber et al. 2023; Merra et al. 2020). However, in our study, microbial diversity was slightly reduced in MD-fed EAE mice, though not significantly different overall.

WD has been associated with increased *Bacteroides*, while MD favors *Prevotella* and *Faecalibacterium prausnitzii* (Jin et al. 2019; Merra et al. 2020). Our results showed higher levels of *Rikenella*,* Odoribacter*, *Peptostreptococcaceae*,* Anaerotruncus*,* Clostridiaceae*, and *Clostridium* in the WD-Lyc group. Conversely, *Dorea*,* Coprococcus*,* Oscillospira*, and *Bilophila* were lower in WD groups. MD increased *Lachnospiraceae* and *Butyricicoccus*, while WD raised *Parabacteroides* and *Bilophila wadsworthi* (Zhu et al. 2020). MD has also been linked to more Bifidobacterium and less dysbiosis genera (Haro et al. 2016), consistent with our finding of higher *Bifidobacterium* in the MD-Lyc group. In MS patients, MD increased *Enterobacteriaceae*,* Akkermansia*, and Blautia but reduced *Faecalibacterium* and *Prevotella*, lowering the *Firmicutes/Bacteroidetes* ratio (Moles et al. 2022); similarly, our MD-Lyc EAE mice had more *Akkermansia*.

Key MD components also shape the microbiota. Dietary fiber boosts SCFA-producing species like *Clostridium leptum* and *Eubacterium rectale*, increases *Ruminococcaceae* and *Lactobacillaceae*, and reduces *Sutterellaceae* and *Coriobacteriaceae* (Berer et al. 2018; Lu et al. 2020). In our study, *Helicobacter*,* Enterobacteriaceae*,* Acinetobacter*, and *Pseudomonas* were lower in the MD-Lyc group. Polyphenols in extra virgin olive oil modify lactic acid bacteria and reduce H. pylori, while n-3 PUFAs lower Firmicutes (Barber et al. 2023). Consistently, MD-Lyc mice had higher levels of *Enterococcus*,* Lactococcus*,* Bacillus*,* Staphylococcus*,* Clostridiales*, and related taxa.

### Limitations

The study has limitations. Monitoring EAE scores longer than 23-day window. Will shed light on possible contributions of MD-Lyc diet in the chronic phase of the disease and its effects on the remyelination process. The mechanisms of CD8^+^ T cell decrease and CD4/CD8 ratio shifting in favor of CD4^+^ T cells need to be examined. Moreover, examining the immune infiltrates in the CNS will also be more guiding in understanding the mechanisms. Lastly, the antioxidant properties of lycopene on CNS infiltrating cells as well as resident microglia can also account for reduced inflammation, the subsequent amelioration in EAE severity, thus mechanisms of this require further study.

## Conclusion

In summary, in this study, the effects of nutritional regimes in which lycopene was combined with Mediterranean diet and Western diet were examined on the course of MS in a mouse model, and it was shown that the combination of lycopene with Mediterranean diet was more effective in alleviating the course of MS. This healing effect developed independently of Treg expansion. The Mediterranean diet and lycopene combination more effectively reduced CD8^+^ T cell numbers in the spleen and lymph node (*p* < 0.05), increased the CD4^+^/CD8^+^ ratio, upregulated IFN-γ production (*p* < 0.05), and downregulated splenic IL-17 production (*p* < 0.05). These changes led to a decrease in EAE clinical scores and caused changes in the intestinal microbiome.

## Electronic Supplementary Material

Below is the link to the electronic supplementary material.

Supplementary Material 1: Supplementary File The experimental procedure started on day– 19, and all groups were given the determined feeds from the beginning to the sacrifice. Naive mice for 28 days and mice in the EAE groups for 43 days were fed with these feeds ad libitum. Mice and remaining feed were weighed daily. Lycopene was administered to all groups every two days for 28 days. On day 0, EAE groups were immunized, and on the ninth day, naïve groups and on the twenty-third day, EAE groups were sacrificed and their organs and tissues were obtained. The number of mice that died in the study was two. Both mice are in the MD-Lyc/EAE group

Supplementary Material 2: Supplementary Tables The feeds used in the study were purchased from the company named Arden Araştırma ve Deney (https://ardenarastirma.com/index.html) in the form of a purified diet and pellets suitable for consumption by mice. Diet compositions were planned similarly to the human diet using the literature. Macronutrient components and contents of the diets are given in Supplementary Tables 1 and Supplementary Table 2. The points considered when preparing diets are as follows: 1.The control diet was adapted from the Western-style diet that is widely consumed today. 2.While the macronutrient composition ratios of all diets were the same, the ratios of the fat types used were determined according to the type of diet. 3.The n-6/n-3 ratio was set at the 2:1 ratio recommended for optimal health in the Mediterranean diet and at the 15–20:1 ratio stated in the literature on the Western diet. 4.The amount of energy coming from sucrose, which represents the rate of free sugar, was calculated in accordance with the recommendations for both diet types. 5.The amount of fiber in the Mediterranean diet was adjusted to be twice that of the Western diet. 6.Olive oil was used as the main fat source in the Mediterranean diet, and corn oil was used in the control diet. 7.While fish oil and flaxseed oil were used in the Mediterranean diet as sources of n-3 fatty acids, fish oil was not added to the control diet and only flaxseed oil was used as a vegetable n-3 source. 8.Butter, which was used as a source of saturated fatty acids, was higher in the control diet. 9.In order to emphasize the polyphenol content of the Mediterranean diet, green tea extract, and resveratrol were added in amounts that would not cause side effects in mice, in accordance with the literature

Supplementary Material 3

## Data Availability

No datasets were generated or analysed during the current study.
