# Supplementary figures and images for: Lycopene Supplemented Mediterranean Diet Ameliorates Experimental Autoimmune Encephalomyelitis (EAE) in Mice and Changes Intestinal Microbiome
Source: J Neuroimmune Pharmacol. 2025 May 5;20(1):50. doi: 10.1007/s11481-025-10212-7 (PMC12052919; doi:10.1007/s11481-025-10212-7)

**Supplementary File 1.** Experimental Procedure


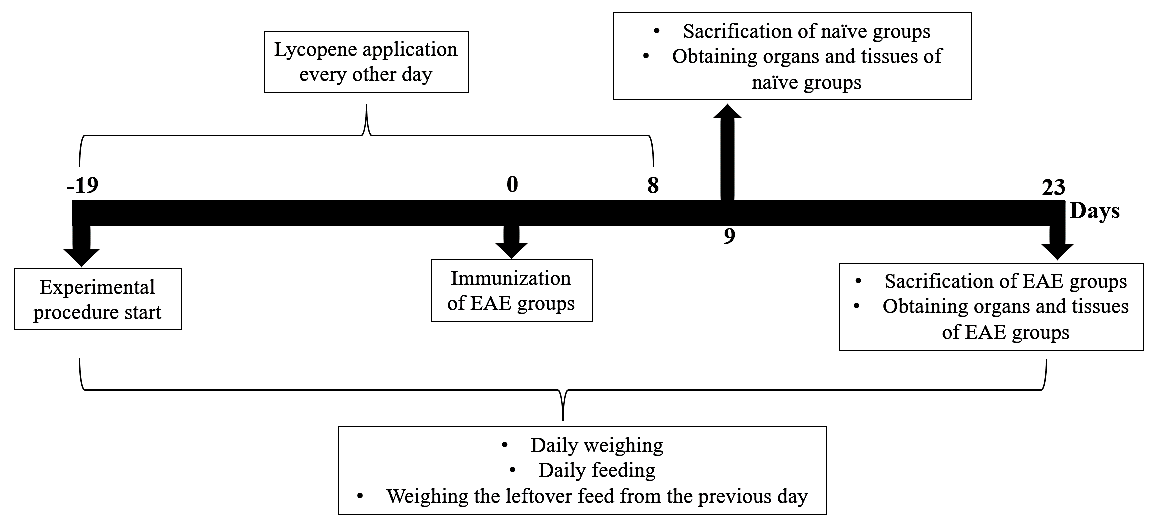

Supplement: Supplementary file 1 — Supplementary Material 1: Supplementary File The experimental procedure started on day– 19, and all groups were given the determined feeds from the beginning to the sacrifice. Naive mice for 28 days and mice in the EAE groups for 43 days were fed with these feeds ad libitum. Mice and remaining feed were weighed daily. Lycopene was administered to all groups every two days for 28 days. On day 0, EAE groups were immunized, and on the ninth day, naïve groups and on the twenty-third day, EAE groups were sacrificed and their organs and tissues were obtained. The number of mice that died in the study was two. Both mice are in the MD-Lyc/EAE group [file 11481_2025_10212_MOESM1_ESM.docx]
