# Supplementary material for: Lycopene Supplemented Mediterranean Diet Ameliorates Experimental Autoimmune Encephalomyelitis (EAE) in Mice and Changes Intestinal Microbiome
Source: J Neuroimmune Pharmacol. 2025 May 5;20(1):50. doi: 10.1007/s11481-025-10212-7 (PMC12052919; doi:10.1007/s11481-025-10212-7)
Supplement: Supplementary file 2 — Supplementary Material 2: Supplementary Tables The feeds used in the study were purchased from the company named Arden Araştırma ve Deney (https://ardenarastirma.com/index.html) in the form of a purified diet and pellets suitable for consumption by mice. Diet compositions were planned similarly to the human diet using the literature. Macronutrient components and contents of the diets are given in Supplementary Tables 1 and Supplementary Table 2. The points considered when preparing diets are as follows: 1.The control diet was adapted from the Western-style diet that is widely consumed today. 2.While the macronutrient composition ratios of all diets were the same, the ratios of the fat types used were determined according to the type of diet. 3.The n-6/n-3 ratio was set at the 2:1 ratio recommended for optimal health in the Mediterranean diet and at the 15–20:1 ratio stated in the literature on the Western diet. 4.The amount of energy coming from sucrose, which represents the rate of free sugar, was calculated in accordance with the recommendations for both diet types. 5.The amount of fiber in the Mediterranean diet was adjusted to be twice that of the Western diet. 6.Olive oil was used as the main fat source in the Mediterranean diet, and corn oil was used in the control diet. 7.While fish oil and flaxseed oil were used in the Mediterranean diet as sources of n-3 fatty acids, fish oil was not added to the control diet and only flaxseed oil was used as a vegetable n-3 source. 8.Butter, which was used as a source of saturated fatty acids, was higher in the control diet. 9.In order to emphasize the polyphenol content of the Mediterranean diet, green tea extract, and resveratrol were added in amounts that would not cause side effects in mice, in accordance with the literature [file 11481_2025_10212_MOESM2_ESM.docx]

**Supplementary Table 1.** Macronutrient components of diets

|  | **Mediterranean Diet (MD)**  **Contribution to daily energy**  **(%)** | **Western Diet (WD)**  **Contribution to daily energy**  **(%)** |
| --- | --- | --- |
| Protein | 15 | 15 |
| Carbohydrate | 50 | 50 |
| Fat | 35 | 35 |
| Saturated fat | 9 | 12 |
| Monounsaturated fat | 18 | 5 |
| Polyunsaturated fat | 8 | 18 |
| n-6/n-3 ratio | 2:1 | 16:1 |

**Supplementary Table 2.** Contents of the diets

|  | **Mediterranean Diet** | | **Western Diet** | |
| --- | --- | --- | --- | --- |
| **Ingredient** | **Amount (g)** | **Energy (kcal)** | **Amount (g)** | **Energy (kcal)** |
| Casein 90 Mash | 140.8 | 563.2 | 99.8 | 399.2 |
| Dried egg white | 9 | 36 | 50 | 200 |
| L-sistein | 3 | 12 | 3 | 12 |
| Corn starch | 0 | 0 | 275.4 | 1101.6 |
| Wheat starch | 380 | 1520 | 0 | 0 |
| Maltodextrin | 40 | 160 | 66.9 | 267.6 |
| Sucrose | 50 | 200 | 120 | 480 |
| Fructose | 20 | 80 | 31.4 | 125.6 |
| Cellulose | 40 | 0 | 20 | 0 |
| Inulin | 10 | 20 | 5 | 10 |
| Olive oil | 81.3 | 731.7 | 22.5 | 202.5 |
| Corn oil | 24.2 | 217.8 | 76.3 | 686.7 |
| Fish oil | 6 | 54 | 0 | 0 |
| Flaxseed oil | 6 | 54 | 4.8 | 43.2 |
| Butter, Anhydrous | 40.7 | 366.3 | 54.1 | 486.6 |
| Di calcium Phosphate | 13 | 0 | 13 | 0 |
| Calcium Carbonate | 5.5 | 0 | 5.5 | 0 |
| Potassium Citrate | 16.5 | 0 | 16.5 | 0 |
| Choline chloride | 2 | 0 | 2 | 0 |
| Green tea extract | 0.1 | 0 | 0 | 0 |
| Resveratrol | 0.1 | 0 | 0 | 0 |
| Vitamin Mix | 10 | 40 | 10 | 40 |
| Mineral mix | 10 | 0 | 10 | 0 |
| Red | 0.05 | 0 | 0.05 | 0 |
| Total protein | 152.8 | 611.2 | 152.8 | 611.2 |
| Total carbohydrate | 500 | 2000 | 503.7 | 2014.8 |
| Total fat | 158.2 | 1423.8 | 157.7 | 1419 |
| Total fiber | 50 | 20 | 25 | 10 |
| **Total** | **909.2** | **4.055** | **886.3** | **4055** |
