# Supplementary material for: Lycopene Supplemented Mediterranean Diet Ameliorates Experimental Autoimmune Encephalomyelitis (EAE) in Mice and Changes Intestinal Microbiome
Source: J Neuroimmune Pharmacol. 2025 May 5;20(1):50. doi: 10.1007/s11481-025-10212-7 (PMC12052919; doi:10.1007/s11481-025-10212-7)
Supplement: Supplementary file 3 — Supplementary Material 3 [file 11481_2025_10212_MOESM3_ESM.docx]

Supplemental Files

Table 3. Immune cells in the spleens of naive mice

|  | **Groups** | | | | **p** |
| --- | --- | --- | --- | --- | --- |
|  | **WD** | **WD-Lyc** | **MD** | **MD-Lyc** |  |
| %CD3+* | 37,97±3,112 | 32,93±2,880 | 38,03±1,483 | 39,07±2,319 | 0,3388 |
| %CD4+* | 60,73±0,6805 | 66,00±1,129 | 62,57±0,5643 | 62,85±1,006 | **0,0037** |
| %CD8+** | 30,95(7,500) | 29,10(7,800) | 29,80(9,800) | 32,35(6,700) | **0,0340** |
| CD4/CD8** | 1,970(0,6029) | 2,235(0,9593) | 2,106(0,8485) | 1,957(0,6406) | **0,0110** |
| %GM-CSF+CD4+** | 3,420(9,510) | 5,020(8,200) | 1,560(10,80) | 0,6300(0,4400) | **0,0140** |
| %IFN-γ+CD4+* | 9,455±1,338 | 15,58±0,9365 | 8,718±1,873 | 8,327±0,3039 | **0,0016** |
| %IL-17A+CD4+* | 1,755±0,3209 | 1,208±0,2085 | 1,332±0,2247 | 0,8433±0,2100 | 0,1034 |
| %IL-22+CD4+** | 6,770(5,320) | 9,110(3,180) | 7,145(3,740) | 6,595(1,600) | **0,0254** |
| %GM-CSF+CD8+* | 7,725±1,571 | 11,73±2,728 | 8,220±2,990 | 3,157±0,2346 | **0,0792** |
| %IFN-γ+CD8+* | 14,89±2,137 | 25,17±4,568 | 15,55±4,098 | 10,38±0,6651 | **0,0307** |
| %IL-17A+CD8+* | 0,8333±0,2088 | 0,5483±0,1319 | 0,6983±0,06750 | 0,4767±0,1241 | 0,3185 |
| %IL-22+CD8+** | 3,865(6,600) | 5,030(6,010) | 2,365(14,02) | 0,9800(1,270) | **0,0230** |
| %Foxp3+* | 10,68±0,5098 | 10,79±1,184 | 11,53±1,099 | 11,13±0,3836 | 0,8981 |
| Foxp3 MFI* | 888,7±20,42 | 893,7±18,43 | 934,3±33,56 | 955,0±6,282 | 0,1268 |
| %CD19+* | 41,05±2,507 | 33,30±1,531 | 39,03±2,053 | 45,40±0,794 | **0,0015** |
| %CD19+CD138+* | 2,050±0,2149 | 2,313±0,3244 | 3,053±0,1938 | 2,143±0,2107 | **0,0328** |
| %CD11b+* | 3,275±0,2431 | 3,335±0,1986 | 2,513±0,08106 | 3,488±0,2958 | **0,0232** |
| %Ly6G+* | 8,890±1,386 | 8,790±1,166 | 15,22±3,200 | 7,100±0,8440 | **0,0317** |
| #CD3+** | 729497(1751561) | 669765(1496369) | 395534(588626) | 664606(3941586) | 0,1378 |
| #CD4+** | 446699(998048 ) | 435016(1038943) | 248932(381639) | 408128(2478606) | 0,1235 |
| #CD8+** | 219343(641022 ) | 197153(315154) | 118163(192881) | 218623(1253253) | 0,1309 |
| #GM-CSF+CD4+** | 12391(30080) | 19399(127212) | 2408(8593) | 2407(15670) | **0,0047** |
| #IFN-γ+CD4+** | 46283(39482) | 66047(208316) | 16380(18343) | 32436(216052) | **0,0022** |
| #IL-17A+CD4+* | 8538±1722 | 7465±2534 | 2776±599,2 | 5918±2428 | 0,2190 |
| #IL-22+CD4+** | 40328(61546) | 37822(112072) | 17500(27538) | 26891(128774) | 0,0990 |
| #GM-CSF+CD8+** | 15115(16344) | 23874(88463) | 6087(4209) | 6665(30850) | **0,0077** |
| #IFN-γ+CD8+** | 36502(31593) | 55001(155831) | 10513(12720) | 22142(95724) | **0,0082** |
| #IL-17A+CD8+** | 2191(3278) | 959,7(2685) | 742,4(1063) | 1075(10130) | 0,2440 |
| #IL-22+CD8+** | 9413(12968) | 9152(34108) | 1800(3903) | 2142(4903) | **0,0029** |
| #Foxp3+** | 54470(125693) | 64437(138446) | 32636(33695) | 46599(240943) | 0,1279 |
| #CD19+** | 1353731(3263177) | 1444005(2053463) | 548286(941053) | 1250172(11097439) | 0,0493 |
| #CD19+CD138+** | 66286(83169) | 100352(66467) | 52568(78272) | 70440(369589) | 0,1929 |
| #CD11b+** | 128526(289932) | 180511(287812) | 44714(72990) | 92588(1236969) | **0,0209** |
| #Ly6G+** | 337967(354518) | 409312(363422) | 243518(217186) | 245312(952023) | 0,0496 |

*One-way ANOVA (X±SEM)

**Kruskal-Wallis (M//IQR)

Table 4. Immune cells in the lymph nodes of naive mice

|  | **Groups** | | | | **p** |
| --- | --- | --- | --- | --- | --- |
|  | **WD** | **WD-Lyc** | **MD** | **MD-Lyc** |  |
| %CD3+* | 82,22±1,042 | 78,72±2,235 | 76,03±2,528 | 76,23±4,768 | 0,4381 |
| %CD4+** | 51,00(6,700) | 48,70(4,500) | 49,20(3,900) | 49,90(6,900) | 0,3408 |
| %CD8+** | 44,05(5,900) | 46,85(7,300) | 45,90(5,300) | 43,90(6,700) | 0,3508 |
| CD4/CD8* | 1,112±0,04369 | 1,059±0,03297 | 1,080±0,02562 | 1,124±0,04088 | 0,5814 |
| %GM-CSF+CD4+* | 0,5300±0,1101 | 1,048±0,2814 | 1,280±0,4423 | 1,878±0,2624 | **0,0341** |
| %IFN-γ+CD4+* | 2,978±0,3880 | 6,652±0,6950 | 5,147±0,9584 | 6,942±0,3826 | **0,0013** |
| %IL-17A+CD4+* | 1,568±0,1199 | 0,7917±0,06052 | 2,128±0,3551 | 1,398±0,1027 | **0,0012** |
| %IL-22+CD4+* | 6,302±0,1926 | 6,388±0,2237 | 8,233±0,7428 | 7,135±0,3147 | **0,0165** |
| %GM-CSF+CD8+** | 0,8950(0,6800) | 2,135(3,530) | 1,145(1,930) | 1,900(2,080) | **0,0035** |
| %IFN-γ+CD8+** | 2,490(1,060) | 6,780(5,800) | 3,625(5,300) | 6,855(3,350) | **0,0012** |
| %IL-17A+CD8+* | 0,3083±0,04246 | 0,1367±0,04551 | 0,6083±0,1216 | 0,2867±0,08849 | **0,0047** |
| %IL-22+CD8+* | 0,9033±0,06946 | 0,7633±0,1251 | 1,037±0,1479 | 1,422±0,1853 | **0,0188** |
| %Foxp3+* | 10,05±0,7161 | 13,12±0,6779 | 8,167±0,4246 | 7,388±0,4315 | **<0,0001** |
| Foxp3 MFI* | 1284±27,80 | 1379±16,91 | 1209±28,88 | 1076±17,84 | **<0,0001** |
| %CD19+* | 27,37±2,737 | 25,08±1,563 | 28,48±2,660 | 27,08±7,379 | 0,9519 |
| %CD19+CD138+* | 1,282±0,2056 | 1,562±0,1405 | 1,773±0,3544 | 1,478±0,3764 | 0,6841 |
| %CD11b+** | 2,570(1,370) | 2,825(2,270) | 1,885(1,830) | 2,480(3,120) | 0,2796 |
| %Ly6G+** | 4,445(6,120) | 6,670(3,880) | 4,715(5,070) | 6,790(14,82) | 0,0973 |
| #CD3+** | 15691(29284) | 42895(75295) | 19314(78867) | 2763(53172) | **0,0476** |
| #CD4+** | 7858(15304) | 21128(34121) | 9138(39781) | 1309(26373) | **0,0477** |
| #CD8+** | 7052(12736) | 19843(37637) | 9048(35949) | 1278(23449) | **0,0437** |
| #GM-CSF+CD4+** | 46,98(126,7) | 176,0(749,5) | 59,09(147,7) | 21,60(429,9) | 0,1371 |
| #IFN-γ+CD4+** | 254,5(466,5) | 1389(2699) | 328,3(1450) | 86,55(1772) | **0,0267** |
| #IL-17A+CD4+** | 125,1(321,7) | 173,0(308,7) | 137,6(919,2) | 21,60(348,1) | 0,1772 |
| #IL-22+CD4+** | 505,1(946,6) | 1366(1961) | 670,9(2900) | 87,73(1954) | 0,0539 |
| #GM-CSF+CD8+** | 70,46(146,0) | 422,3(1312) | 106,1(325,3) | 30,82(480,7) | **0,0157** |
| #IFN-γ+CD8+** | 176,6(287,3) | 1483(2604) | 328,8(1013) | 80,71(1820) | **0,0149** |
| #IL-17A+CD8+** | 21,69(45,60) | 24,29(99,22) | 28,67(223,2) | 4,802(49,24) | 0,1965 |
| #IL-22+CD8+** | 65,04(124,8) | 127,2(378,5) | 76,97(311,2) | 16,23(347,0) | 0,2941 |
| #Foxp3+** | 1445(2432) | 4723(10024) | 1592(4914) | 260,7(3531) | **0,0106** |
| #CD19+** | 13865(11247) | 35289(61559) | 15987(34353) | 7802(32385) | **0,0185** |
| #CD19+CD138+* | 796,5±187,5 | 2398±551,8 | 1138±224,2 | 649,0±339,2 | **0,0094** |
| #CD11b+** | 1463(1289) | 3675(11872) | 1185(2212) | 444,4(3861) | **0,0208** |
| #Ly6G+** | 2136(6227) | 9312(17389) | 3466(5159) | 1453(10699) | **0,0417** |

*One-way ANOVA (X±SEM)

**Kruskal-Wallis (M//IQR)

Table 5. Immune cells in the spleens of EAE mice

|  | **Groups** | | | | **p** |
| --- | --- | --- | --- | --- | --- |
|  | **WD** | **WD-Lyc** | **MD** | **MD-Lyc** |  |
| %CD3+* | 23,46±1,664 | 23,37±1,252 | 30,37±1,416 | 18,35±1,173 | **<0,0001** |
| %CD4+* | 55,97±0,5665 | 54,78±0,5804 | 59,20±0,6458 | 58,26±0,4792 | **<0,0001** |
| %CD8+* | 28,78±0,4289 | 31,51±0,7523 | 27,98±0,7303 | 26,24±0,7176 | **<0,0001** |
| CD4/CD8* | 1,953±0,04778 | 1,753±0,05673 | 2,138±0,07570 | 2,238±0,07496 | **<0,0001** |
| %GM-CSF+CD4+** | 6,125(6,420) | 5,900(3,960) | 6,535(5,110) | 9,080(13,22) | **0,0050** |
| %IFN-γ+CD4+* | 21,96±0,9629 | 23,96±0,6876 | 24,59±0,8556 | 28,24±0,8476 | **0,0001** |
| %IL-17A+CD4+* | 3,658±0,2431 | 2,689±0,1233 | 2,635±0,1063 | 2,402±0,1107 | **<0,0001** |
| %IL-22+CD4+* | 12,10±0,2517 | 11,43±0,2782 | 11,40±0,2716 | 11,81±0,2157 | 0,1768 |
| %GM-CSF+CD8+** | 3,750(14,79) | 3,960(4,080) | 4,530(9,450) | 7,805(10,89) | **0,0015** |
| %IFN-γ+CD8+* | 56,98±2,554 | 61,21±1,373 | 61,06±2,500 | 67,59±2,060 | **0,0181** |
| %IL-17A+CD8+* | 21,82±1,210 | 18,36±0,5200 | 19,24±1,049 | 16,54±0,9076 | **0,0046** |
| %IL-22+CD8+** | 1,450(5,660) | 1,080(2,050) | 1,035(2,080) | 1,790(4,050) | **0,0250** |
| %Foxp3+* | 14,81±0,5773 | 14,81±0,2981 | 15,20±0,3838 | 15,53±0,3691 | 0,5970 |
| Foxp3 MFI** | 999,5(378,0) | 977,5(218,0) | 1080(367,0) | 1025(222,0) | 0,0764 |
| %CD19+* | 40,95±2,455 | 38,68±1,917 | 36,67±1,387 | 39,00±2,732 | 0,5562 |
| %CD19+CD138+* | 3,535±0,1620 | 3,610±0,2579 | 3,498±0,2788 | 3,131±0,2074 | 0,5215 |
| %CD11b+* | 5,823±0,3212 | 6,171±0,2142 | 6,122±0,1853 | 6,501±0,1995 | 0,2957 |
| %Ly6G+* | 7,538±0,2753 | 7,634±0,2931 | 8,728±0,2522 | 8,243±0,3721 | **0,0193** |
| #CD3+* | 1385143 ±161989 | 1745067±340920 | 2463841±281963 | 1272050±291335 | **0,0172** |
| #CD4+* | 778476±94167 | 951144±185522 | 1455347 ±163501 | 741416±168090 | **0,0081** |
| #CD8+* | 395887±44523 | 552129±105862 | 691564±83191 | 330809±73926 | **0,0143** |
| #GM-CSF+CD4+* | 51175±7621 | 57901±12308 | 106634±15456 | 68793±14338 | **0,0140** |
| #IFN-γ+CD4+* | 169496±20894 | 223351±41849 | 363344±43559 | 204364±44539 | **0,0045** |
| #IL-17A+CD4+* | 29258±4885 | 26480±5266 | 37133±3401 | 18762±5182 | 0,0751 |
| #IL-22+CD4+* | 94713±11953 | 108846±22087 | 166221±18520 | 86025±19289 | **0,0150** |
| #GM-CSF+CD8+** | 13583(70431) | 12303(41743) | 32945(113830) | 22503(47731) | 0,0621 |
| #IFN-γ+CD8+* | 225339±27250 | 332620±63359 | 431600±58824 | 218566±45572 | **0,0155** |
| #IL-17A+CD8+* | 87069±11360 | 102166±20637 | 127849±13459 | 57635±16363 | **0,0306** |
| #IL-22+CD8+** | 4936(26684) | 3686(13156) | 7935(19902) | 4987(8843) | 0,4973 |
| #Foxp3+* | 147270±20942 | 225219±40421 | 263704±31079 | 168693±34065 | 0,0548 |
| #CD19+* | 4731094 ±643346 | 5362169±1014153 | 5340665±667977 | 4555667±982261 | 0,8670 |
| #CD19+CD138+** | 390337(979718) | 398852(862915) | 481213(1137257) | 274631(580188) | 0,4948 |
| #CD11b+* | 859909±129647 | 1035030±197067 | 1121950±167113 | 1049734±250338 | 0,7737 |
| #Ly6G+* | 1142840 ±193669 | 1295930±250109 | 1619248±226823 | 1238624±248413 | 0,4830 |

*One-way ANOVA (X±SEM)

**Kruskal-Wallis (M//IQR)

Table 6. Immune cells in the lymph nodes of EAE mice

|  | **Groups** | | | | **p** |
| --- | --- | --- | --- | --- | --- |
|  | **WD** | **WD-Lyc** | **MD** | **MD-Lyc** |  |
| %CD3+* | 35,85±1,196 | 36,03±2,230 | 45,03±1,928 | 35,81±2,288 | **0,0022** |
| %CD4+* | 51,48±0,7184 | 50,15±0,4327 | 54,14±0,7694 | 55,08±0,7646 | **<0,0001** |
| %CD8+* | 32,95±0,5694 | 32,39±0,5557 | 28,68±0,6630 | 27,89±0,6036 | **<0,0001** |
| CD4/CD8* | 1,568±0,03558 | 1,553±0,02632 | 1,901±0,05799 | 1,986±0,06157 | **<0,0001** |
| %GM-CSF+CD4+* | 6,511±0,5113 | 7,408±0,2899 | 8,204±0,6734 | 9,651±0,5279 | **0,0014** |
| %IFN-γ+CD4+** | 12,05(32,91) | 9,550(5,550) | 12,40(36,30) | 10,55(6,050) | **0,0013** |
| %IL-17A+CD4+** | 2,675(1,770) | 2,245(2,050) | 2,350(1,230) | 3,170(14,45) | **0,0003** |
| %IL-22+CD4+* | 2,888±0,08642 | 2,855±0,07031 | 3,006±0,04528 | 3,173±0,1740 | 0,1267 |
| %GM-CSF+CD8+** | 0,09450(0,2550) | 0,1700(0,3040) | 0,1000(0,2600) | 0,1200(0,3780) | 0,1380 |
| %IFN-γ+CD8+** | 0,9500(4,470) | 0,8500(1,710) | 0,9450(7,650) | 0,9800(1,210) | 0,8908 |
| %IL-17A+CD8+* | 21,98±1,099 | 20,26±0,8344 | 21,98±0,8378 | 28,14±2,379 | **0,0014** |
| %IL-22+CD8+** | 0,000(0,000) | 0,000(0,03100) | 0,000(0,000) | 0,000(0,03400) | 0,5154 |
| %Foxp3+* | 14,67±0,3970 | 13,89±0,5122 | 14,58±0,3646 | 15,00±0,6305 | 0,4280 |
| Foxp3 MFI* | 2029±30,42 | 1924±61,12 | 1851±46,22 | 1586±65,18 | **<0,0001** |
| %CD19+* | 51,26±2,295 | 48,78±2,445 | 34,38±1,491 | 43,03±2,252 | **<0,0001** |
| %CD19+CD138+** | 1,830(4,340) | 1,590(3,400) | 1,580(0,9800) | 1,690(2,660) | 0,6095 |
| %CD11b+* | 3,628±0,1310 | 3,614±0,1381 | 3,908±0,1116 | 4,739±0,2965 | **0,0001** |
| %Ly6G+* | 7,855±0,4570 | 7,679±0,2300 | 8,490±0,2859 | 10,09±0,5482 | **0,0004** |
| #CD3+* | 395744±95799 | 438241±59860 | 616755±118332 | 245277±60650 | **0,0512** |
| #CD4+* | 201768±48616 | 218231±28781 | 331968±62763 | 135073±34091 | **0,0405** |
| #CD8+* | 132796±32559 | 139396±17378 | 179070±36213 | 67283±16472 | 0,0673 |
| #GM-CSF+CD4+* | 12355±2975 | 16120±2061 | 25895±5021 | 12708±3078 | **0,0287** |
| #IFN-γ+CD4+** | 21874(80220) | 21564(27870) | 32057(160897) | 12197(35837) | **0,0163** |
| #IL-17A+CD4+* | 5012±1107 | 4836±656,6 | 7354±1095 | 7088±2533 | 0,4461 |
| #IL-22+CD4+* | 5935±1494 | 6152±769,7 | 9869±1833 | 4325±1136 | **0,0507** |
| #GM-CSF+CD8+* | 141,2±30,13 | 223,0±43,03 | 160,9±39,02 | 89,76±25,14 | 0,0948 |
| #IFN-γ+CD8+** | 1050(6184) | 1137(2583) | 1655(16629) | 476,6(2198) | **0,0117** |
| #IL-17A+CD8+* | 30605±8220 | 27819±3453 | 39448±8188 | 19759±4670 | 0,2468 |
| #IL-22+CD8+** | 0,000(0,000) | 0,000(36,83) | 0,000(0,000) | 0,000(30,72) | 0,5220 |
| #Foxp3+* | 43015±8662 | 53881±9276 | 58804±14216 | 26026±6086 | 0,1514 |
| #CD19+** | 750693(1667030) | 832855(1923895) | 543801(2033822) | 395967(753606) | 0,0591 |
| #CD19+CD138+* | 33659±9125 | 28723±3155 | 30086±5597 | 16085±2748 | 0,2239 |
| #CD11b+* | 72533±14769 | 88968±13183 | 98160±23725 | 53782±11550 | 0,2995 |
| #Ly6G+* | 156584±35478 | 190436±30550 | 204308±41336 | 118788±25284 | 0,3355 |

*One-way ANOVA (X±SEM)

**Kruskal-Wallis (M//IQR)
